# Formulation of an Efficient O (*M* ^4^)-Scaling Explicitly Correlated MP2-F12 Correction by Combining Numerical Quadrature with Density Fitting and CABS-RI

**DOI:** 10.1021/acs.jctc.5c01874

**Published:** 2026-03-02

**Authors:** Lars Urban, Henryk Laqua, Travis H. Thompson, Christian Ochsenfeld

**Affiliations:** † Chair of Theoretical Chemistry, Department of Chemistry, University of Munich (LMU), D-81377 Munich, Germany; ‡ Max Planck Institute for Solid State Research, D-70569 Stuttgart, Germany

## Abstract

We present a novel approach that combines numerical quadrature with density fitting and CABS-RI for the evaluation of exchange-type intermediates in RI-MP2-F12 theory, rigorously reducing the formal and practical scaling of the total correction from $O(M^{5})$ to $O(M^{4})$ . Our new hybrid NQ/DF/CABS-RI ansatz is based directly on our previously developed NQ/CABS-RI method for the efficient evaluation of 6c3e integrals [Urban, L.; Laqua, H; Thompson, T. H.; Ochsenfeld, C. *J. Chem. Theory Comput.* **2024**, *20*, 3706–3718] and extends this approach to the optimized computation of products of 4c2e integrals. In this framework, the main exchange-type intermediates V , X , and B are reformulated, resulting in more compact expressions, increased shared computations, and fewer CABS-RI insertions. We introduce efficient algorithms that cover all exchange-type contributions, including advantageous batching of integrals. Benchmarks show that NQ/DF/CABS-RI achieves mean errors below 0.01 kcal/mol for noncovalent interaction and isomerization energies already with small to modest grid sizes, while the numerical precision can be adjusted to balance computational cost. Empirical scaling was determined using linear glycine chains, demonstrating the expected $O(M^{4})$ behavior for the rate-determining steps, with the remaining exchange-type expressions scaling nearly linearly. Compared with an idealized DF/CABS-RI implementation, our approach achieves speedups of roughly one order of magnitude for the most expensive steps with virtually no loss of numerical accuracy. Systems with strongly delocalized electronic structures benefit particularly. For a nanotube with 168 carbon atoms, the computational time for the most demanding expressions is reduced from 9.97 to 1.25 days, bringing the cost much closer to that of conventional DF-MP2. At present, NQ/DF/CABS-RI achieves efficient $O(M^{4})$ scaling, and further cost reductions are anticipated through the introduction of integral screening based on Cholesky orbitals, which will be explored in future work.

## Graphical abstract

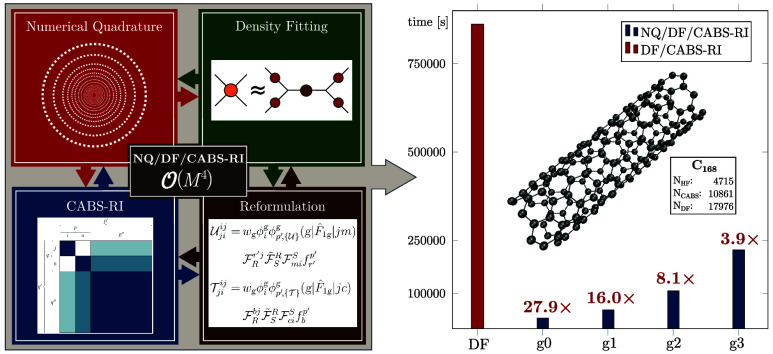

## 1 Introduction

To this day, the highly accurate and efficient computation of electronic structures of many-body systems remains one of the central challenges in quantum chemistry and physics. While the number of correlated electrons and their corresponding orbitals in small systems remains manageable for steep-scaling high-level methods, their practical applicability to larger systems is severely limited. In particular, accurately describing short-range dynamic correlation significantly increases the computational cost. Conventional correlation methods require a prohibitively large set of basis functions to properly capture the cusp behavior of the exact wave function near the coalescence of two electrons due to the $\frac{1}{r_{12}}$ singularity of the Coulomb operator.^1^ Alternatively, ansätze that explicitly incorporate the interelectronic distance *r*_12_ into their wave function description overcome this issue, allowing for a significantly reduced orbital space. First introduced for practical calculations by Hylleraas in 1929 with his pioneering study of the helium atom,^2^ the general idea of including terms explicitly depending on *r*_12_ has led to various approaches, e.g., variational Hylleraas-configuration interaction (Hylleraas-CI),^3–6^ explicitly correlated Gaussian (ECG) wave function approaches,^7–12^ or transcorrelated (TC) methods.^13–34^

Among these methods, the family of explicitly correlated F12 corrections has emerged as the most popular and widely applied, being typically suited for larger systems than most alternative approaches while significantly accelerating the convergence with respect to the size of the one-electron basis. Its success traces back to the seminal work of Kutzelnigg and Klopper on so-called R12 corrections,^35–37^ which introduced explicitly coupled two-electron terms (geminals) into the wave function description. To handle the resulting high-dimensional integrals, such as six-center three-electron (6c3e) and eight-center four-electron (8c4e) integrals, they proposed applying the Resolution-of-the-Identity (RI) technique to decompose them into tractable sums of four-center two-electron (4c2e) integrals. While the original R12 ansatz exhibited intrinsic limitations due to the use of a linear *r*_12_ correlation factor, which is not optimally suited to describe short-range electron–electron correlation, Ten-no^38^ succeeded in reproducing the correct electron–electron cusp behavior by introducing a flexible exponentially decaying correlation factor, giving rise to what is today known as F12 theory. Following this development, F12 approaches continued to evolve, incorporating several key improvements, most notably Valeev’s complementary auxiliary basis set (CABS)^39^ method for an advantageous partitioning of orbital spaces, as well as the efficient use of density fitting (DF) techniques^40,41^ to drastically reduce the computational prefactor by decomposing 4c2e integrals into three-center-two-electron (3c2e) and two-center-two-electron (2c2e) integrals. Today, DF/CABS-RI F12 approaches are well-established tools, offering an accurate and robust treatment of complex systems with a wide range of corrections for different quantum-chemical methods, such as in perturbation theory,^42–49^ coupled-cluster theory,^50–58^ the random-phase-approximation (RPA),^59–61^ multireference methods,^62–69^ in density functional theory (DFT) design,^70,71^ and corresponding gradients.^72–75^

Most of these approaches rely on the standard DF/CABS-RI framework to evaluate the required integrals, which often remains computationally demanding. In fact, for explicitly correlated corrections to second-order Mo̷ller–Plesset perturbation theory (MP2-F12), the cost of the F12 correction can significantly exceed that of the underlying MP2 calculation. A less common alternative in this regard is three-dimensional numerical quadrature (NQ),^76–79^ which was originally introduced to F12 theory for the accurate evaluation of multielectron integrals.^80,81^

We have recently proposed a novel and highly efficient atomic orbital-based ansatz for exchange-type six-center three-electron (6c3e) F12 integrals, combining numerical quadrature (NQ) with CABS-RI and distance-dependent integral screening.^82^ By expressing the most computationally demanding term in RI-MP2-F12 theory in the form of such a 6c3e integral, this approach enables substantial reductions in computational effort, yielding significant speedups and a linear-scaling evaluation. However, only certain exchange-type terms in RI-MP2-F12 theory can be efficiently transformed into 6c3e integrals, while the remaining contributions must still be treated as products of four-center two-electron (4c2e) integrals. For direct-type terms, the standard DF/CABS-RI framework remains the most efficient choice, offering an asymptotic $O(M^{4})$ scaling with respect to the system size *M*. In contrast, exchange-type terms evaluated using DF/CABS-RI alone retain a steeper $O(M^{5})$ scaling.

In the following, we introduce a new approach that combines density fitting and CABS-RI with numerical quadrature to efficiently reduce the computational cost and rigorously lower the formal scaling of the exchange-type product of two 4c2e integrals to $O(M^{4})$ . For each major exchange-type intermediate, we identify the most efficient evaluation strategy, employing fewer CABS-RI insertions and reformulated terms. Furthermore, we present efficient algorithms with optimized memory demand for computing exchange-type contributions for all operators involved in RI-MP2-F12 theory, ultimately lowering the overall scaling of the F12 correction to $O(M^{4})$ —below the standard $O(M^{5})$ scaling of DF-MP2.

## 2 Theory

Throughout this work, we use the orbital spaces defined in Table 1.

**Table 1:** Summary of Orbital Spaces and Indexing Conventions

orbital space	indices
AO Hartree–Fock space	μ, ν, λ, σ, ···
AO complementary auxiliary space	μ″, ν″, λ″, σ″, ···
combined AO HF/CABS space ({μ} ∪ {μ″})	μ′, ν′, λ′, σ′, ···
geminal space	*x*, *y*, *w*, *z*
MO active occupied space	*i*, *j*
MO occupied space	*k*, *l*
MO virtual space	*a*, *b*, *c*, *d*
MO occupied + virtual space ({*i*} ∪ {*a*})	*p*, *q*, *r*, *s*
MO complementary auxiliary space	*p*″, *q*″, *r*″, *s*″
combined MO HF/CABS space ({*p*} ∪ {*p*″})	*p*′, *q*′, *r*′, *s*′
density-fitting space	*P*, *Q*, *R*, *S*

To maintain the readability of the occurring F12 intermediates, we employ an implicit summation convention for indices that appear repeatedly and exclusively within a single expression on one side of an equation (e.g., in energy expressions or AO basis contractions). Explicit summation signs are included only where they are necessary for clarity or to emphasize a specific transformation. Conversely, equations defining the individual elements of a tensor or operator (such as the definitions of the major F12 intermediates) are understood such that indices appearing on both sides of the equation are fixed and do not imply summation. To represent the various types of electronic integrals, we employ both Dirac and Mulliken (chemical) notations. For a general two-body operator Ô _12_, a four-center two-electron (4c2e) integral over spatial orbitals can be written in either form:

$$\underset{\mathrm{Dirac}}{\underset{︸}{\left\langle ij|{Ô}_{12}|kl\right\rangle }}=\underset{\mathrm{Mulliken}}{\underset{︸}{\left(ik|{Ô}_{12}|jl\right)}}=\iint \phi_{i}^{*}(r_{1})\phi_{j}^{*}(r_{2}){Ô}_{12}\phi_{k}(r_{1})\phi_{l}(r_{2})dr_{1}dr_{2}$$ 1

In the following, we discuss the efficient use of numerical quadrature (NQ)^80–84^ and its combination with density fitting (DF) techniques^40,41^ and CABS-RI^39^ for the evaluation of exchange-type intermediates in F12 theory, with particular emphasis on their application within a modified version of the closed-shell MP2-F12­(3*C) correction.^43^ In this context, the acronym RI (as in RI-MP2-F12) explicitly denotes the use of an MO completeness insertion via CABS-RI, while DF is used exclusively for the approximation of two-electron integrals. We begin with a brief review of the standard 3*C correction, also referred to as 3C­(FIX). The correction employs the correlation factor ${\mathrm{F̂}}_{12}$ , the Coulomb operator ${ĝ}_{12}$ , and corresponding products, defined as

$${\mathrm{F̂}}_{12}=\frac{1}{\gamma }e^{-\gamma r_{12}}$$ 2

$${ĝ}_{12}=\frac{1}{r_{12}}$$ 3

$${\mathrm{F̂}}_{12}{ĝ}_{12}=\frac{1}{\gamma }e^{-\gamma r_{12}}\cdot \frac{1}{r_{12}}$$ 4

$${\mathrm{F̂}}_{12}^{2}=\frac{1}{\gamma^{2}}e^{-2\gamma r_{12}}$$ 5

where *r*_12_ is the interelectronic distance. In the 3*C ansatz, the strong orthogonality projector ${\mathrm{Q̂}}_{12}$ takes the form

$${\mathrm{Q̂}}_{12}=(1-{ô}_{1})(1-{ô}_{2})(1-{\mathrm{v̂}}_{1}{\mathrm{v̂}}_{2})$$ 6

where ô_*n*_ and v̂_*n*_ are projectors onto the occupied and virtual orbital spaces for electron *n*, respectively, enforcing orthogonality with respect to double excitations within the explicitly correlated geminal space. Furthermore, the rational generator *Ŝ*_*xy*_ ^80^ ensures the simultaneous fulfillment of the *s*- and *p*-wave coalescence conditions, given by

$${Ŝ}_{xy}=\frac{3}{8}+\frac{1}{8}{\mathrm{L̂}}_{xy}$$ 7

where the permutation operator ${\mathrm{L̂}}_{xy}$ exchanges the spatial coordinates ***r*** while preserving the spin coordinates σ of two electrons:

$${\mathrm{L̂}}_{xy}\phi_{x}(r_{1},\sigma_{1})\phi_{y}(r_{2},\sigma_{2})=\phi_{x}(r_{2},\sigma_{1})\phi_{y}(r_{1},\sigma_{2})$$ 8

The computationally demanding $O(M^{6})$ variational optimization of geminal amplitudes is avoided by employing the fixed-amplitude ansatz

$$c_{ij}^{xy}=\delta_{x}^{i}\delta_{y}^{j}$$ 9

which restricts the explicitly correlated geminal space to the Hartree–Fock occupied orbitals, leading to the widely used diagonal, orbital-invariant formulation. In combination with the generalized Brillouin condition (GBC) and the extended Brillouin condition (EBC), which neglect Fock matrix elements between the virtual/occupied and RI auxiliary orbital spaces, the explicitly correlated F12 correction to the MP2 energy in the 3*C approximation takes the form^47^

EF123*C=54Vijij−14Vjiij+732Bjiij+132Bjiij−732(Xilij fjl+Xkjij fik)−132(Xliij fjl+Xjkij fik) 10

The evaluation of the three primary exchange-type intermediates $V_{ji}^{ij}$ , $X_{ji}^{ij}$ , and $B_{ji}^{ij}$ clearly dominates the overall computational cost (see Section 4.2) and thus, their efficient evaluation is of central importance for a high-performance implementation. These intermediates are defined as

$$V_{ji}^{ij}=\langle ij|{\mathrm{F̂}}_{12}{\mathrm{Q̂}}_{12}{ĝ}_{12}|ji\rangle$$ 11

$$X_{ji}^{ij}=\langle ij|{\mathrm{F̂}}_{12}{\mathrm{Q̂}}_{12}{\mathrm{F̂}}_{12}|ji\rangle$$ 12

$$B_{ji}^{ij}=\langle ij|{\mathrm{F̂}}_{12}{\mathrm{Q̂}}_{12}({\mathrm{f̂}}_{1}+{\mathrm{f̂}}_{2}){\mathrm{Q̂}}_{12}{\mathrm{F̂}}_{12}|ji\rangle$$ 13

where ${\mathrm{f̂}}_{1}$ and ${\mathrm{f̂}}_{2}$ denote Fock operators, and *f*_*i*_^*k*^ and *f*_*j*_^*l*^ represent Fock matrix elements, which reduce to the orbital energies ϵ_*i*_ and ϵ_*j*_ when canonical molecular orbitals are used. For further details on the derivation of these intermediates, as well as on other F12 approaches to which the techniques described in the following can be applied, the reader is referred to the literature.^85,86^

The conventional evaluation of $V_{ji}^{ij}$ , $X_{ji}^{ij}$ , and $B_{ji}^{ij}$ using only CABS-RI and density fitting exhibits an unfavorable $O\left(M^{5}\right)$ scaling,^45^ as standard density fitting techniques reduce only the computational prefactor without improving the formal scaling behavior. However, combining these methods with numerical quadrature enables a reduction to $O\left(M^{4}\right)$ for the arising products of 4c2e integrals, as discussed for a generic case in Section 2.1. Subsequently, we present an alternative, CABS-RI free evaluation of the $V_{ji}^{ij}$ and $X_{ji}^{ij}$ exchange-type intermediates based on six-center three-electron (6c3e) integrals (Section 2.2), and provide a detailed strategy for evaluating the complicated $B_{ji}^{ij}$ intermediate with fewer approximations, simplified expressions, and a reduced number of CABS-RI insertions (Section 2.3). Finally, in Section 2.4, we outline efficient implementation strategies for all intermediates, focusing on minimizing computational cost and memory usage through the reuse of subintermediates and the consolidation of shared computational steps across different expressions.

### 2.1 Decomposition Techniques

Several approaches have been introduced to handle the complicated intermediates V , X , and B , whose approximation-free evaluation leads to high-dimensional expressions involving up to eight-center four-electron (8c4e) integrals, which are infeasible to compute in practice. In particular, the efficient use of the Resolution-of-the-Identity (RI) approximation has been key to the success of R12/F12 methods as it enables the decomposition of high-dimensional integrals into products of lower-dimensional ones. Here, the identity operator 1̂ on the one-electron function space can be exactly resolved for electron *n* as

$${\mathrm{\alpha ̂}}_{n}g(...,r_{n},...)=\sum_{\alpha }\alpha (r_{n})\left(\int \alpha (r_{n})g(...,r_{n},...)dr_{n}\right)$$ 14

where α̂_*n*_ denotes the formally exact projector onto a complete orthonormal basis. To construct an appropriate approximate projector, one can obtain a large orthogonal set of auxiliary basis functions {*P*_*o*_}, e.g., via Löwdin orthogonalization

$$P_{o}(r)=[S^{-1/2}{]}_{Q}^{P}Q(r)$$ 15

with ***S*** being the overlap matrix in the auxiliary basis {*P*}. The projector α̂_*n*_ can then be approximated as

$${\mathrm{\alpha ̂}}_{n}\approx |P_{o})(P_{o}|=|P)(PQ{)}^{-1}(Q|$$ 16

where we use the shorthand notation (*PQ*)^−1^ = [***S*** ^–1^]_*Q*_^*P*^. The most widely used RI scheme in F12 theory today is Valeev’s complementary auxiliary basis set (CABS) approach,^39^ in which the Resolution-of-the-Identity is performed in the combined orbital space *p*′, formed by the union of the Hartree–Fock orbitals *p* and the CABS basis functions *p*″. Whereas CABS-RI plays a key role in the evaluation of integrals in most parts of F12 theory, especially for the direct terms in RI-MP2-F12­(3*C), we restrict its use for exchange-type contributions to the evaluation of the B intermediate, as it involves a large orbital space and entails significant computational cost. However, in this context, it can be used efficiently to decompose expressions into exchange-type six-center three-electron (6c3e) integrals. We have recently demonstrated that these 6c3e integrals can be evaluated with high efficiency and accuracy using numerical quadrature in combination with distance-dependent integral screening, leading to a linear scaling computation of the most expensive term in RI-MP2-F12 theory.^82^ A typical approach to decompose an unspecified molecular orbital 6c3e integral using numerical quadrature is given by

$${\mathrm{WY}}_{kij}^{ijk}=\langle ijk|{Ŵ}_{12}{Ŷ}_{23}|kij\rangle \approx w_{g}\phi_{j}^{g}\phi_{i}^{g}(g|{Ŵ}_{1g}|ik)(g|{Ŷ}_{1g}|kj)$$ 17

where ${Ŵ}_{12}$ = *W*(| ***r***_1_ – ***r***_2_|) and *Ŷ*_23_ = *Y* (| ***r***_2_ – ***r***_3_|) represent generic distance-dependent operators occurring in F12 theory, *g* and *w*_*g*_ denote discrete grid points and their corresponding weights, and ϕ_*i*_^*g*^ describes the *i*-th molecular orbital evaluated at grid point *g*. The 3c1e MO integrals (*g*| ${Ŵ}_{1g}$ |*ik*) with ${Ŵ}_{1g}$ = *W*(| ***r***_1_ – ***r***_*g*_|), are computed via an $O\left(M^{4}\right)$ scaling AO to MO transformation of the 3c1e AO integrals

$$(g|{Ŵ}_{1g}|\mu \nu )=\int \chi_{\mu }(r_{1})\chi_{\nu }(r_{1}){Ŵ}_{1g}dr_{1}$$ 18

The energy contribution of the 6c3e integral is then obtained via three at most formally $O\left(M^{3}\right)$ scaling steps

$$\mathrm{step}\;1:,W_{k}^{g}=\phi_{i}^{g}(g|{Ŵ}_{1g}|ik),(N_{g}N_{k}N_{i})$$ 19

$$\mathrm{step}\;2:,Y_{k}^{g}=\phi_{j}^{g}(g|{Ŷ}_{1g}|kj),(N_{g}N_{k}N_{i})$$ 20

$$\mathrm{step}\;3:,E_{\left\{{\mathrm{WY}}_{kij}^{ijk}\right\}}=w_{g}W_{k}^{g}Y_{k}^{g},,(N_{g}N_{k})$$ 21

with the formal time complexity for the steepest scaling step given in parentheses. Although the AO to MO transformation of some of the 3c1e AO integrals can not be avoided for an efficient computation of $B_{ji}^{ij}$ , the evaluation of 6c3e exchange-type integrals in the pure AO regime can still be beneficial, as shown in the upcoming sections, resulting in

⟨ijk|Ŵ12Ŷ23|kij⟩≈wgcμj χμgcνi χνgcλicσk(g|Ŵ1g|λσ)cδkcεj(g|Ŷ1g|δε) 22

⁣=wgPμεPνλPσδ χμgχνg(g|Ŵ1g|λσ)(g|Ŷ1g|δε) 23

with MO coefficients *c*, density matrix elements *P*_μν_ = ∑_*i*_ *c*_μ*i*_ *c*_ν*i*_ for closed-shell systems, and χ_μ_^*g*^ denoting the μ-th atomic orbital evaluated at grid point *g*, respectively. Here, eq 23 is best computed via a stepwise $O\left(M^{3}\right)$ scaling evaluation

step1:⁣χ®εg=Pμε χμg⁣(NgNμ2) 24

$$\mathrm{step}\;2:,W_{\sigma }^{g}={\overset{®}{\chi }}_{\lambda }^{g}(g|{Ŵ}_{1g}|\lambda \sigma ),(N_{g}N_{\mu }^{2})$$ 25

$$\mathrm{step}\;3:,Y_{\delta }^{g}={\overset{®}{\chi }}_{\varepsilon }^{g}(g|{Ŷ}_{1g}|\delta \varepsilon ),(N_{g}N_{\mu }^{2})$$ 26

$$\mathrm{step}\;4:,E_{\left\{{\mathrm{WY}}_{kij}^{ijk}\right\}}=w_{g}P_{\sigma \delta }W_{\sigma }^{g}Y_{\delta }^{g},(N_{g}N_{\mu }^{2})$$ 27

The advantages of an AO-based approach lie in the ability to combine the overlap decay of the basis functions, the sparsity of the density matrix, and the operator decay into an efficient, distance-dependent integral screening for the evaluation of the 3c1e AO integrals, as described in detail in Section 2 of ref 82. Moreover, the use of block-sparse matrix algebra (BSMA)^87^ in the contractions of eqs 24–27 enables a reduction of computational scaling to linear by leveraging the inherent sparsity of the involved quantities.

Unfortunately, not all exchange-type terms can be expressed in the form of 6c3e integrals but instead decompose into products of two four-center two-electron (4c2e) integrals. In previous work,^43,45^ such terms have predominantly been treated using density fitting techniques, as exemplified by

$$W_{kl}^{ij}Y_{ji}^{kl}=\langle ij|{Ŵ}_{12}|kl\rangle \langle kl|{Ŷ}_{12}|ji\rangle \approx W_{P}^{ik}{\mathrm{W̃}}_{Q}^{P}W_{jl}^{Q}Y_{R}^{kj}{Ỹ}_{S}^{R}Y_{li}^{S}$$ 28

with

$$Y_{R}^{kj}=(kj|{Ŷ}_{12}|R)$$ 29

$${Ỹ}_{S}^{R}=[Y^{-1}{]}_{S}^{R}$$ 30

$$Y_{S}^{R}=(R|{Ŷ}_{12}|S)$$ 31

While a purely density-fitting-based approach substantially lowers the computational prefactor, the formal scaling of the overall computation remains $O\left(M^{5}\right)$ . Here, we propose an alternative ansatz that combines density fitting with numerical quadrature, thereby reducing the formal scaling to $O\left(M^{4}\right)$ . Specifically, we express the product of two 4c2e integrals as

$$\langle ij|{Ŵ}_{12}|kl\rangle \langle kl|{Ŷ}_{12}|ji\rangle \approx w_{g}\phi_{i}^{g}\phi_{k}^{g}(g|{Ŵ}_{1g}|jl)Y_{R}^{kj}{Ỹ}_{S}^{R}Y_{li}^{S}$$ 32

which can again be stepwise computed via

$$\mathrm{step}\;1:,{Ỹ}_{li}^{R}={Ỹ}_{S}^{R}Y_{li}^{S},(N_{P}^{2}N_{k}N_{i})$$ 33

$$\mathrm{step}\;2:,{Ỹ}_{lg}^{R}=\phi_{i}^{g}{Ỹ}_{li}^{R},(N_{g}N_{P}N_{k}N_{i})$$ 34

$$\mathrm{step}\;3:,Y_{R}^{gj}=\phi_{k}^{g}Y_{R}^{kj},(N_{g}N_{P}N_{k}N_{i})$$ 35

$$\mathrm{step}\;4:,Y_{gjl}={Ỹ}_{lg}^{R}Y_{R}^{gj},(N_{g}N_{P}N_{k}N_{i})$$ 36

$$\mathrm{step}\;5:,E_{\left\{{\mathrm{WY}}_{ji}^{ij}\right\}}=w_{g}(g|{Ŵ}_{1g}|jl)Y_{gjl},(N_{g}N_{k}N_{i})$$ 37

The choice of which electron is represented as molecular orbitals on the grid, and consequently the placement of the numerical quadrature within the integral product, is crucial for achieving maximum efficiency in the evaluation, particularly for integrals involving some form of CABS-RI. Optimal performance is typically achieved when the largest orbital space is represented as MO on a grid and an early contraction is performed. This approach is especially advantageous for integrals involving additional multiple orbital spaces spanning Fock or exchange matrix elements,^49^ as it allows an early contraction of the largest indices with a formal scaling of $O\left(M^{3}\right)$ .

### $V_{ji}^{ij}$ - and $X_{ji}^{ij}$ -Intermediate

2.2

Both intermediates, $V_{ji}^{ij}$ (eq 11) and $X_{ji}^{ij}$ (eq 12), share a similar structure and can be evaluated using the same decomposition scheme. For $X_{ji}^{ij}$ , we assume that canonical molecular orbitals are used for the Fock matrix elements *f*_*i*_^*k*^ and *f*_*j*_^*l*^, yielding the corresponding orbital energies ϵ_*i*_ and ϵ_*j*_. In contrast to the conventional approach, which relies on an approximate form of the strong orthogonality projector,

$${\mathrm{Q̂}}_{12}^{\mathrm{approx}}=1-{\mathrm{p̂}}_{1}{\mathrm{p̂}}_{2}-{ô}_{1}{\mathrm{p̂}}_{2}^{\prime \prime }-{\mathrm{p̂}}_{1}^{\prime \prime }{ô}_{2}$$ 38

we instead employ the exact operator as defined in eq 6. The exact expression for both exchange-type intermediates is then given by

$$V_{ji}^{ij}={\mathrm{FG}}_{ji}^{ij}+F_{kl}^{ij}G_{ji}^{kl}-F_{ab}^{ij}G_{ji}^{ab}-\langle ijk|{\mathrm{F̂}}_{12}{ĝ}_{23}|kij\rangle -\left\langle jik|{\mathrm{F̂}}_{12}{ĝ}_{23}|kji\right\rangle$$ 39

$$X_{ji}^{ij}={\mathrm{FF}}_{ji}^{ij}+F_{kl}^{ij}F_{ji}^{kl}-F_{ab}^{ij}F_{ji}^{ab}-\langle ijk|{\mathrm{F̂}}_{12}{\mathrm{F̂}}_{23}|kij\rangle -\left\langle jik|{\mathrm{F̂}}_{12}{\mathrm{F̂}}_{23}|kji\right\rangle$$ 40

leading for each intermediate to a combination of two identical true 6c3e exchange-type integrals, one 4c2e integral, and two products of 4c2e integrals. For $V_{ji}^{ij}$ , the arising integrals are best decomposed as follows:

FGjiij≈wgPμσPνλ χμgχνg(g|F̂1gĝ1g|λσ) 41

$$F_{kl}^{ij}G_{ji}^{kl}\approx F_{P}^{ik}{\mathrm{F̃}}_{Q}^{P}F_{jl}^{Q}w_{g}\phi_{k}^{g}\phi_{j}^{g}(g|{ĝ}_{1g}|li)$$ 42

$$F_{ab}^{ij}G_{ji}^{ab}\approx F_{P}^{ia}{\mathrm{F̃}}_{Q}^{P}F_{jb}^{Q}w_{g}\phi_{a}^{g}\phi_{j}^{g}(g|{ĝ}_{1g}|bi)$$ 43

⟨ijk|F̂12ĝ23|kij⟩≈wgPμεPνλPσδ χμgχνg(g|F̂1g|λσ)(g|ĝ1g|δε) 44

where the AO formalism is applied when it is most efficient, leading to a formal scaling of $O(N_{g}N_{\mu }^{2})$ for eqs 41 and 44, while the combined MO-based terms in eqs 42 and 43 scale as $O(N_{g}N_{P}N_{p}N_{i})$ . The decomposition of $X_{ji}^{ij}$ leads to analogously scaling terms,

FFjiij≈wgPμσPνλ χμgχνg(g|F̂1g2|λσ) 45

$$F_{kl}^{ij}F_{ji}^{kl}\approx F_{P}^{ik}{\mathrm{F̃}}_{Q}^{P}F_{jl}^{Q}w_{g}\phi_{k}^{g}\phi_{j}^{g}(g|{\mathrm{F̂}}_{1g}|li)$$ 46

$$F_{ab}^{ij}G_{ji}^{ab}\approx F_{P}^{ia}{\mathrm{F̃}}_{Q}^{P}F_{jb}^{Q}w_{g}\phi_{a}^{g}\phi_{j}^{g}(g|{\mathrm{F̂}}_{1g}|bi)$$ 47

⟨ijk|F̂12F̂23|kij⟩≈wgPμεPνλPσδ χμgχνg(g|F̂1g|λσ)(g|F̂1g|δε) 48

where the operators on the grid for both intermediates are defined as

$${\mathrm{F̂}}_{1g}=\frac{1}{\gamma }e^{-\gamma r_{1g}}$$ 49

$${ĝ}_{1g}=\frac{1}{r_{1g}}$$ 50

$${\mathrm{F̂}}_{1g}{ĝ}_{1g}=\frac{1}{\gamma }e^{-\gamma r_{1g}}\cdot \frac{1}{r_{1g}}$$ 51

$${\mathrm{F̂}}_{1g}^{2}=\frac{1}{\gamma^{2}}e^{‐2\gamma r_{1g}}$$ 52

with *r*_1*g*_ = | ***r***_1_ – ***r***_*g*_|. In eqs 42 and 43, the 4c2e integrals involving classical Coulomb operator ${ĝ}_{12}$ are decomposed via numerical quadrature, rather than those involving the correlation factor ${\mathrm{F̂}}_{12}$ . This eliminates the need to access additional 3c2e and 2c2e integrals during the computation, as the required 3c1e integrals for ĝ _12_ are already used in eq 44, thereby lowering the memory demand. Beyond the reduction in formal scaling, a key benefit of using the exact form of *Q̂*_12_ is that the evaluation remains entirely CABS-RI free, eliminating associated errors and overhead.

### $B_{ji}^{ij}$ -Intermediate

2.3

Among all intermediates in RI-MP2-F12 theory, $B_{ji}^{ij}$ (eq 13) stands out as the most intricate and computationally intensive, due to the incorporation of Fock operators into the integral expression in conjunction with the projectors in *Q̂*_12_ (eq 6). In this context, the symmetry of ${\mathrm{F̂}}_{12}$ and *Q̂*_12_ with respect to the electron labels is exploited to treat ${\mathrm{f̂}}_{1}$ and ${\mathrm{f̂}}_{2}$ on equal footing, allowing us to write

⟨ij|F̂12Q̂12 f̂1Q̂12F̂12|ji⟩=⟨ji|F̂12Q̂12 f̂2Q̂12F̂12|ij⟩ 53

so that only *Q̂*_12_ ${\mathrm{f̂}}_{1}$ *Q̂*_12_ needs to be considered. Inserting the definition of *Q̂*_12_ leads to the following distinct combinations of operators and projectors:

 f̂1− f̂1ô1− f̂1ô2 f̂1ô1ô2− f̂1v̂1v̂2−ô1 f̂1ô1 f̂1ô1ô1 f̂1ô2−ô1 f̂1ô1ô2ô1 f̂1v̂1v̂2−ô2 f̂1ô2 f̂1ô1ô2 f̂1ô2−ô2 f̂1ô1ô2ô2 f̂1v̂1v̂2ô1ô2 f̂1−ô1ô2 f̂1ô1−ô1ô2 f̂1ô2ô1ô2 f̂1ô1ô2−ô1ô2 f̂1v̂1v̂2−v̂1v̂2 f̂1v̂1v̂2 f̂1ô1v̂1v̂2 f̂1ô2−v̂1v̂2 f̂1ô1ô2v̂1v̂2 f̂1v̂1v̂2 54

Fortunately, we can leverage the idempotency of projectors, the commuting behavior of operators, and the identities

ônv̂n=v̂nôn=ôn f̂nv̂n=v̂n f̂nôn=0 55

which, together with the removal of canceling terms, allows for the reduction of eq 54 to the following form:

 f̂1− f̂1ô1− f̂1ô2 f̂1ô1ô2− f̂1v̂1v̂2−ô1 f̂1ô1 f̂1ô1ô1ô2 f̂1−ô1 f̂1ô1ô200000000000−v̂1v̂2 f̂1000v̂1 f̂1v̂1v̂2 56

Overall, *Q̂*_12_ ${\mathrm{f̂}}_{1}$ *Q̂*_12_ can thus be expressed as

Q̂12 f̂1Q̂12= f̂1− f̂1ô2+T̂(− f̂1ô1+ f̂1ô1ô2− f̂1v̂1v̂2)+ô1 f̂1ô1−ô1 f̂1ô1ô2+v̂1 f̂1v̂1v̂2 57

where the linear operator *T̂* introduces the transpose, e.g., T̂ f̂1ô1ô2 = ${\mathrm{f̂}}_{1}{ô}_{1}{ô}_{2}$ + ô1ô2 f̂1, which combines identical terms due to the symmetry of the resulting integrals. The approximation-free evaluation of the integrals arising from the operator combinations in eq 57 results in complex multielectron integrals, including up to eight-center four-electron integrals, along with nonstandard Fock matrix elements. Previous work avoided these by applying various CABS-RI insertions, splitting the resulting integrals exclusively into products of 4c2e integrals. This strategy yields a set of (sub)­intermediates, which appears to be the most efficient approach for handling direct-type integrals.

However, for the exchange-type $B_{ji}^{ij}$ intermediate, we developed a more advanced approach that requires fewer CABS-RI insertions. Our novel formalism decomposes certain operator and projector combinations from eq 57 into 6c3e integrals that can be highly efficiently evaluated, and moreover, leads to a more efficient use of DF + NQ for the remaining products of 4c2e integrals. In this context, we exploit the fact that, for ${\mathrm{f̂}}_{1}$ , the nuclear attraction *v̂* and the mean-field Coulomb operator *ĵ* commute with the correlation factor ${\mathrm{F̂}}_{12}$ , which enables the formulation of the well-known commutator relation^43,45^

F̂12 f̂1F̂12=12[[F̂12,t̂1],F̂12]−F̂12k̂1F̂12+12(( f̂1+k̂1)F̂122+F̂122( f̂1+k̂1)) 58

where the term involving the kinetic energy operator *t̂* can be further decomposed using the product rules for the Laplacian Δ_1_ and the gradient operator ∇_1_ as

$$\left[[{\mathrm{F̂}}_{12},{\mathrm{t̂}}_{1}],{\mathrm{F̂}}_{12}]=(\nabla_{1}{\mathrm{F̂}}_{12}\cdot \nabla_{1}{\mathrm{F̂}}_{12}\right)$$ 59

This expression can then be evaluated in the AO picture using NQ as

⟨ij|(∇1F̂12·∇1F̂12)|ji⟩≈γ2wgPμσPνλ χμgχνg(g|F̂1g2|λσ) 60

The last two terms in eq 58 are identical and can be transformed using a single full-RI insertion. In combination with NQ, this yields

$$\langle ij|({\mathrm{f̂}}_{1}+{\mathrm{k̂}}_{1}){\mathrm{\alpha ̂}}_{1}{\mathrm{F̂}}_{12}^{2}|ji\rangle \approx w_{g}P_{\mu \lambda }P_{\nu \sigma }P_{\varepsilon^{\prime }\delta^{\prime }}\chi_{\mu }^{g}\chi_{\varepsilon^{\prime }}^{g}(g|{\mathrm{F̂}}_{1g}^{2}|\nu \lambda ){\left(f+k\right)}_{\sigma }^{\delta^{\prime }}$$ 61

where most of the computational effort for both eqs 60 and 61 is shared with expressions occurring in the $X_{ji}^{ij}$ intermediate, scaling as $O(N_{g}N_{\mu }^{2})$ and $O(N_{g}N_{\mu^{\prime }}N_{\mu })$ , respectively. The operator combinations *k̂*_1_ (result of eq 58), ${\mathrm{f̂}}_{1}$ ô_1_, and ô_1_ ${\mathrm{f̂}}_{1}$ ô_1_ give rise to integrals that are, or can be, transformed into 6c3e integrals through full-RI insertions α̂*′*, which are most efficiently evaluated within the AO formalism as follows:

$${\mathrm{k̂}}_{1}\to {\mathrm{\alpha ̂}}_{1}^{\prime }{\mathrm{k̂}}_{1}{\mathrm{\alpha ̂}}_{1}^{\prime }:\langle ijp^{\prime }|{\mathrm{F̂}}_{12}{\mathrm{F̂}}_{23}|q^{\prime }ij\rangle k_{q^{\prime }}^{p^{\prime }}\approx w_{g}P_{\mu \lambda }P_{\nu \sigma }P_{\delta^{\prime }\gamma^{\prime }}P_{\varepsilon^{\prime }\zeta^{\prime }}\chi_{\mu }^{g}\chi_{\nu }^{g}(g|{\mathrm{F̂}}_{1g}|\lambda \delta^{\prime })(g|{\mathrm{F̂}}_{1g}|\sigma \varepsilon^{\prime })k_{\gamma^{\prime }}^{\zeta^{\prime }}$$ 62

$${\mathrm{f̂}}_{1}{ô}_{1}\to {\mathrm{\alpha ̂}}_{1}^{\prime }{\mathrm{f̂}}_{1}{ô}_{1}:\langle ijp^{\prime }|{\mathrm{F̂}}_{12}{\mathrm{F̂}}_{23}|lij\rangle f_{l}^{p^{\prime }}\approx w_{g}P_{\mu \lambda }P_{\nu \sigma }P_{\delta \gamma }P_{\varepsilon^{\prime }\zeta^{\prime }}\chi_{\mu }^{g}\chi_{\nu }^{g}(g|{\mathrm{F̂}}_{1g}|\lambda \delta )(g|{\mathrm{F̂}}_{1g}|\sigma \varepsilon^{\prime })f_{\gamma }^{\zeta^{\prime }}$$ 63

ô1 f̂1ô1:⟨ijk|F̂12F̂23|lij⟩flk≈wgPμλPνσPδγPεζχμgχνg(g|F̂1g|λδ)(g|F̂1g|σε)fγζ 64

Besides the exchange and Fock matrix elements, which are contracted in a final step, eqs 63 and 64 span only subspaces of eq 62. Consequently, most parts of these three expressions can be evaluated simultaneously at no additional computational cost, while retaining the overall $O(N_{g}N_{\mu^{\prime }}^{2})$ formal scaling, as analyzed and efficiently implemented for eq 62 in ref 82.

The remaining operator combinations ${\mathrm{f̂}}_{1}$ ô_2_, ${\mathrm{f̂}}_{1}$ ô_1_ô_2_, ô_1_ ${\mathrm{f̂}}_{1}$ ô_1_ô_2_, ${\mathrm{f̂}}_{1}$ v̂_1_v̂_2_, and v̂_1_ ${\mathrm{f̂}}_{1}$ v̂_1_v̂_2_ are evaluated as products of 4c2e integrals in the MO representation, requiring at most a double full-RI insertion in combination with DF and NQ. This yields the following relations:

 f̂1ô2→α̂1′ f̂1α̂1′ô2:Fp′mij fr′p′Fjir′m≈wgϕigϕp′g(g|F̂1g|jm)FRr′jF̃SRFmiS fr′p′ 65

 f̂1ô1ô2→α̂1′ f̂1ô1ô2:Fp′mij flp′Fjilm≈wgϕigϕp′g(g|F̂1g|jm)FRljF̃SRFmiS flp′ 66

ô1 f̂1ô1ô2:Fkmij flkFjilm≈wgϕigϕkg(g|F̂1g|jm)FRljF̃SRFmiS flk 67

 f̂1v̂1v̂2→α̂1′ f̂1v̂1v̂2:Fp′cij fbp′Fjibc≈wgϕigϕp′g(g|F̂1g|jc)FRbjF̃SRFciS fbp′ 68

v̂1 f̂1v̂1v̂2:Facij fbaFjibc≈wgϕigϕag(g|F̂1g|jc)FRbjF̃SRFciS fba 69

where eqs 66 and 67 share the same subspaces as eq 65, and eq 69 as eq 68. These terms can be computed simultaneously by scaling the respective subspaces once, which is easily achieved by adjusting the *p*′-th MO on grid ϕ_*p′*_^*g*^, accordingly. We can thus formulate the following intermediates

Ujiij=wgϕigϕp′,{U}g(g|F̂1g|jm)FRr′jF̃SRFmiS fr′p′⁣(NgNPNp′Ni) 70

Tjiij=wgϕigϕp′,{T}g(g|F̂1g|jc)FRbjF̃SRFciS fbp′⁣(NgNPNaNi) 71

with $\phi_{p^{\prime },\{U\}}^{g}$ and $\phi_{p^{\prime },\{T\}}^{g}$ as scaled MO’s on the grid. $U_{ji}^{ij}$ represents the most computationally demanding intermediate in the total explicitly correlated correction. It is the only exchange-type term for which the approximated complete space *p′* cannot be contracted in an $O\left(M^{3}\right)$ evaluation, but instead requires a $O(N_{g}N_{P}N_{p^{\prime }}N_{i})$ scaling contraction. Fortunately, this step can be carried out early in the computation, effectively removing *p′* from subsequent evaluations. Generally, the structure of $U_{ji}^{ij}$ and $T_{ji}^{ij}$ is more naturally suited to a homogeneous evaluation with numerical quadrature and density fitting than the conventional ansatz of separating B into intermediates optimized for pure density fitting, leading to greater reuse of intermediate computations.

### 2.4 Implementation

In the following, we present an efficient implementation of the previously derived terms, focusing on computational performance and memory usage. Careful optimization is key to achieving efficiency and avoiding significant computational overhead. We provide a step-by-step breakdown of the implementation, including the formal scaling of all performance-critical operations, with the most expensive step highlighted. For each operator, we present dedicated algorithms yielding the corresponding energy contributions, starting with Algorithm 1, which addresses the ${\mathrm{F̂}}_{12}{ĝ}_{12}$ operator and introduces the key concepts. Here, we assume that the density matrix *P* and a molecular grid (MG)^88^ are provided by the underlying code base. 
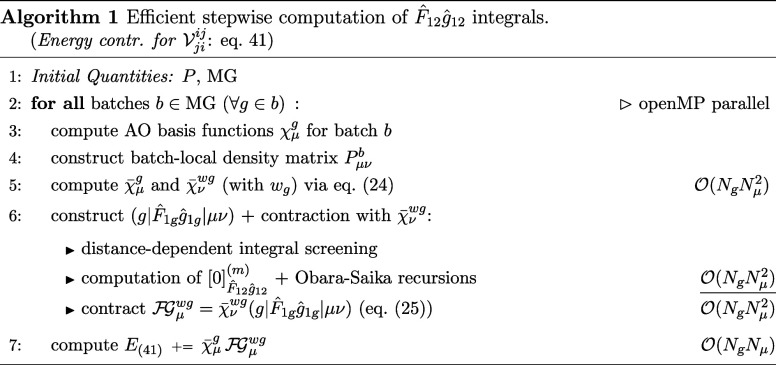
Algorithm 1 closely follows the procedure used in seminumerical exact-exchange methods within Hartree–Fock and hybrid density functional theory, e.g., sn-LinK,^89^ and builds on their underlying infrastructure and conceptual framework. Energy contributions are evaluated in a batchwise manner by partitioning the molecular grid into suitable subsets *b*, each containing at most 64 grid points *g* to maintain consistency with the more memory-intensive evaluations of other operators. For completeness, the formal scaling of the most important steps is explicitly given. Only the significant atomic orbitals χ_μ_^*g*^ (AOs) are constructed for each batch *b* and contracted with the corresponding batch-local density matrix *P*_μν_^*b*^. We additionally exploit operator sparsity and apply a highly efficient distance-dependent integral screening scheme during the construction of the 3c1e integrals (*g*|*F̂*_1*g*_ ĝ _1*g*_|μν), as detailed in ref 82, Section 2.3. Together with the decay of basis function overlaps and the asymptotic sparsity of the batch-local density matrix for systems with a significant HOMO–LUMO gap, this enables an efficient, linear-scaling evaluation of eq 41. Here, the construction of the primitive ${\left[0\right]}_{{\mathrm{F̂}}_{12}{ĝ}_{12}}^{\left(m\right)}.$ Ten-no integrals remains the primary bottleneck and is even more expensive than the corresponding Obara–Saika^90^ recursion scheme for medium- to large-sized systems.
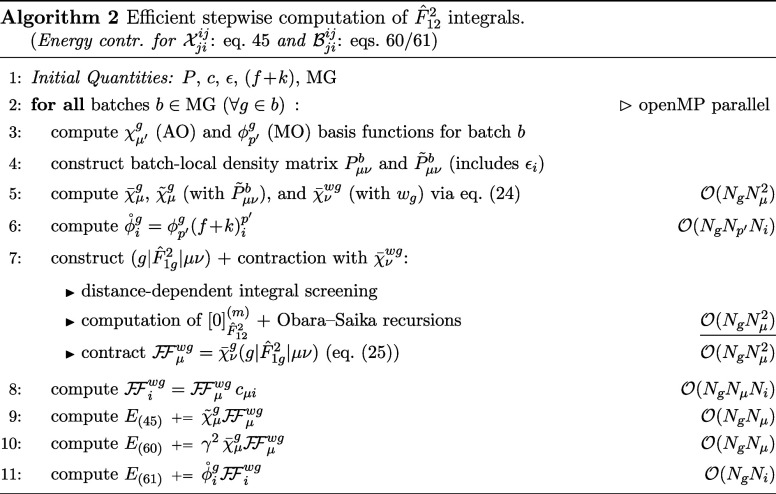


The evaluation of terms involving the ${\mathrm{F̂}}_{12}^{2}$ operator in Algorithm 2 closely mirrors that in Algorithm 1, with the primary distinction being the incorporation of orbital energies ϵ, which leads to adapted local density matrices *P̃*_μν_^*b*^. Otherwise, the evaluation follows the same general structure. The additional contributions in eqs 60 and 61 arise from the commutator relation in eq 58 and may be omitted depending on the chosen level of approximation. For eq 60, the final energy contribution is simply scaled by γ^2^, where γ is the exponent of the correlation factor ${\mathrm{F̂}}_{12}$ . The (*f* + *k*) matrices are introduced by transforming ${FF}_{\mu }^{wg}$ into the molecular orbital basis. Algorithm 2 likewise enables a highly efficient, low-scaling implementation with asymptotically linear computational complexity using only numerical quadrature for the evaluation of the occurring 4c2e integrals.
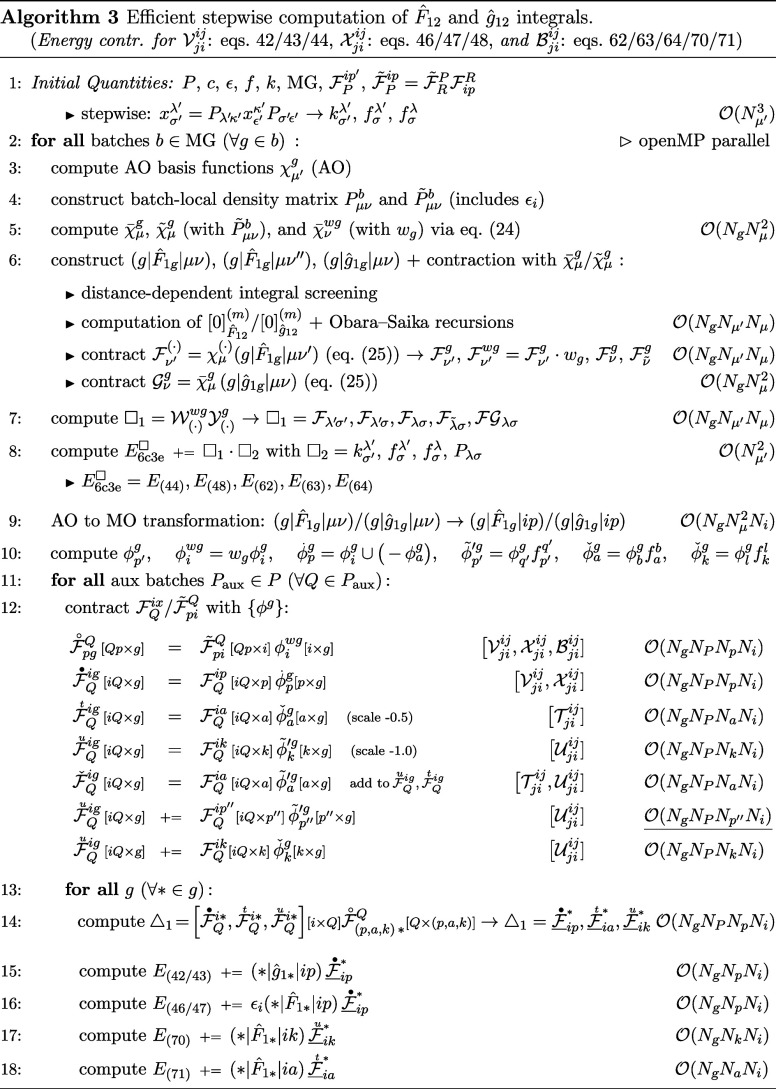


The efficient evaluation of all integrals involving either one or both of the ${\mathrm{F̂}}_{12}$ and ${ĝ}_{12}$ operators is outlined in Algorithm 3, based on a hybrid NQ/DF/CABS-RI scheme that combines atomic and molecular orbital representations. All 6c3e integrals are treated entirely in the AO basis, as exemplified in detail for eq 62 in ref 82, which enables a linear scaling evaluation of these contributions. The remaining products of 4c2e integrals appearing in the intermediates $V_{ji}^{ij}$ , $X_{ji}^{ij}$ , and $B_{ji}^{ij}$ are handled in the MO basis. While a purely AO-based implementation of these products is, in principle, possible and would yield asymptotically linear scaling due to the decay of both electron density and operator contributions, this approach suffers from a substantially larger computational overhead. In contrast, any MO-based treatment, including localized approaches, benefits from the reduced dimensionality of the occupied MO space (number of electrons) compared to the HF AO space (number of basis functions) and can further exploit restrictions such as the frozen core approximation, making it more practical for basically all feasible systems. If multiple similar contractions differ only by the orbital space or operator, we report the formal scaling based on the most expensive one among them.

The AO part of Algorithm 3 largely follows the strategies employed in Algorithms 1 and 2, differing primarily by additional contractions for the 6c3e integrals. The use of the commutator relation only slightly increases the computational cost, as the contraction of the combined 3c1e integrals with the exchange matrix elements *k*_σ′_^λ′^ scales merely as $O(N_{\mu^{\prime }}^{2}N_{b})$ , with *N*_*b*_ denoting the number of batches, and represents just one of several contributing terms.

Regarding the MO part of Algorithm 3, the construction of the MO 3c1e integrals is summarized in a single step. However, for a memory-efficient implementation, the different subspaces and operators are treated separately, and the AO to MO transformation is carried out subsequently and in batches of 16 numerical grid points. This approach ensures that only one subset of AO 3c1e integrals needs to be kept in memory at a given time. In this context, one major advantage of following our new evaluation through $U_{ji}^{ij}$ and $T_{ji}^{ij}$ is that it avoids the use of $\left(g|{\mathrm{F̂}}_{1g}|{ip}^{\prime \prime }\right)$ integrals, thus bypassing the corresponding $O(N_{g}N_{\mu^{\prime \prime }}N_{\mu }N_{i})$ scaling of the AO to MO transformation and the associated memory requirements. The next step involves constructing molecular orbitals for each batch of grid points, which are scaled by a factor or contracted with multiple orbital spaces spanning the Fock matrix elements. This enables an efficient contraction over the approximated complete space index *p′*, incurring a computational cost of only $O(N_{g}N_{p^{\prime }}^{2})$ .

All subsequent evaluations scale as $O\left(M^{4}\right)$ and may require up to *N*_*g*_ *N*_*P*_ *N*_*p*_ of memory per batch. To alleviate this demand, we recommend introducing an additional batching scheme for the auxiliary basis functions used in density fitting, with each auxiliary batch *P*_aux_ comprising 16 auxiliary functions *Q*. This choice is motivated by the fact that the auxiliary index is the only one shared across all storage-intensive intermediates as well as both uncontracted and contracted three-center two-electron integrals, thereby avoiding inefficient matrix-vector products during contraction. For the latter, we applied an optimized reordering scheme by organizing the data into auxiliary batches. The recommended storage layout is indicated in square brackets, for example, [*xy* × *z*], with *x* as the fastest-changing index and *z* as the leading one. This format ensures that, in column-major storage, the data can be directly interpreted as a *xy* × *z* matrix. Further contraction with transposed molecular orbitals $\left\{\phi^{g}\right\}$ via matrix–matrix multiplication yields the intermediates ${\overset{◦}{F}}_{pg}^{Q},\;{\overset{•}{F}}_{Q}^{ig},\;{\overset{u}{F}}_{Q}^{ig}$ , and ${\overset{t}{F}}_{Q}^{ig}$ , all of which have g as their leading index. In this step, the remaining full-RI index *p′* is contracted out, representing the most computationally demanding operation in the entire explicitly correlated code, with a total formal scaling over all batches of $O(N_{g}N_{P}N_{p^{\prime }}N_{i})$ . The resulting intermediates can then be used to compute the final contributions for each grid point within the batch via additional matrix–matrix multiplications, yielding the desired energies upon contraction with the respective MO 3c1e integrals.

Due to the increased memory requirements of the hybrid AO and MO evaluation in Algorithm 3, we generally recommend smaller grid batch sizes of 64 or 128, which only marginally affect the performance of the distance-dependent integral screening.^82,91^ With additional batching over the auxiliary basis functions, the memory footprint per grid batch scales only quadratically as $O(N_{p^{\prime }}N_{i})$ , although with a considerable prefactor. For all parts of the algorithm involving densely populated intermediates, we recommend using high-performance matrix algebra routines (BLAS-3), such as Intel MKL.^92^ To further reduce the number of significant elements, one possible strategy could be to enhance sparsity in the MO representation by introducing localized orbitals. To preserve the correspondence between the Fock matrix elements *f* and the orbital energies ϵ, however, some occupied indices would need to remain in the canonical form during contractions. The additional sparsity introduced by localization could potentially be exploited in conjunction with the intrinsic overlap and operator sparsity of the AO 3c1e and 3c2e F12 integrals.

## 3 Computational Details

All calculations presented in this work were carried out with a development version of our FermiONs++ program package.^93–96^ Preliminary SCF calculations were converged to within 10^–7^ in the DIIS commutator norm,^97,98^ defined as ||**FPS** – **SPF**||. Hartree–Fock and F12-type Fock matrix elements were evaluated using sn-LinK^89,99^ with a gm­[5/3] multigrid, and RI-J^100^ in combination with the cc-pVXZ-JKfit basis^101^ (X = D, T, Q), as described in ref 49. The explicitly correlated F12 corrections employed a fixed Slater-type geminal (STG) correlation factor,^38,81^ defined as ${\mathrm{F̂}}_{12}=\frac{1}{\gamma }\exp(-\gamma r_{12})$ with γ = 1.3, together with the cc-pVXZ-F12 basis set family.^102–104^ The corresponding complementary auxiliary basis sets, cc-pVXZ-F12/OptRI+,^105^ and the density-fitting sets, cc-pVXZ-F12/MP2fit,^106^ were used accordingly (X = D, T, Q). All numerical quadrature computations employed highly optimized numerical grids^88^ (denoted as gX) with a grid point threshold of 10^–5^, as summarized in Table 2. Integral kernels and the corresponding FermiONs++ binary were compiled with the Intel Compiler 19.1.0 ^107^ using the optimization flags -Ofast and -march = native, enabling AVX-512 instructions for optimal efficiency. Performance was assessed on two AMD EPYC 9334 processors (64 cores, 2.5 GHz base clock, with a theoretical maximum single-core boost frequency of 3.91 GHz) with SMT disabled to reduce the effective memory footprint per thread. Basis set superposition errors (BSSE) were corrected via a mixed scheme employing counterpoise uncorrected and corrected values.^108^

**Table 2:** Summary of the Grid Layout for the Carbon Atom, Separated into Inner, Medium, and Outer Regions without Grid Point Screening

grid	*n*_rad_	*n*_ang_ (inner/medium/outer)	*n*_tot,C_
g0	15	14/38/74	916
g1	20	14/50/110	1646
g2	25	26/74/194	3372
g3	35	38/110/302	7040
g4	45	50/194/434	13,012
g7	65	110/434/1454	57,284

## 4 Results

In the following section, we compare our novel hybrid NQ/DF/CABS-RI ansatz with the conventional DF/CABS-RI approach in terms of both numerical precision and performance. Precision is assessed against virtually exact reference data obtained with an extensive g7 grid (57,284 points per carbon atom) and without distance-dependent screening of the 3c1e AO integrals (ϑ_NQ_ = 0) using NQ/DF/CABS-RI. We evaluate noncovalent interaction (NCI) and isomerization energies using cc-pVXZ-F12 (X = D, T, Q) basis-set combinations (HF/CABS-RI/DF) for the S22 ^109^ and ISO34 ^110^ benchmark sets, and provide cc-pVDZ-F12 results for the L7 set,^111^ including larger systems (Section 4.1). Additional results for the S66 set,^112^ showing similar trends, as well as a detailed comparison of absolute energies for the L7 set, illustrating the impact of the DF/CABS-RI and NQ/DF/CABS-RI ansatz on the total correlation energy, are provided in the Supporting Information (SI). To investigate efficiency, we separate the performance analysis into the computation of the 4c2e integrals (Algorithms 1 and 2) and of the remaining intermediates (Algorithm 3), considering different grid sizes and screening thresholds (Section 4.2). For the first two algorithms, we demonstrate near-linear scaling using glycine chains,^113^ in line with previous findings. For Algorithm 3, we further report timings for daptomycin and a carbon nanotube to illustrate real-world performance. To ensure fair comparison across program packages, reference DF/CABS-RI timings are based on the FLOP counts of the rate-determining steps divided by the peak BLAS-3 matrix–matrix multiplication (DGEMM) efficiency, excluding memory allocation and copying. Finally, we emphasize that no form of MO integral screening is applied throughout this analysis.

### 4.1 Precision

Figure 1a–f illustrates the effect of employing NQ/DF/CABS-RI instead of DF/CABS-RI (AO 3c2e integral screening via IPB^91^ with screening threshold ϑ_DF_ = 10^–9^) on the precision of DF-MP2 + F12 NCI and isomerization energies for the S22 (a,c,e) and ISO34 (b,d,f) benchmark sets using double-, triple-, and quadruple-ζ basis set combinations. The plots show mean absolute errors (MAE), maximum absolute errors (MAX), and mean absolute errors normalized to the average DF-MP2 + F12 reference energy (MAE/AVG) for different AO 3c1e integral screening thresholds ϑ_NQ_.

**Figure 1:**
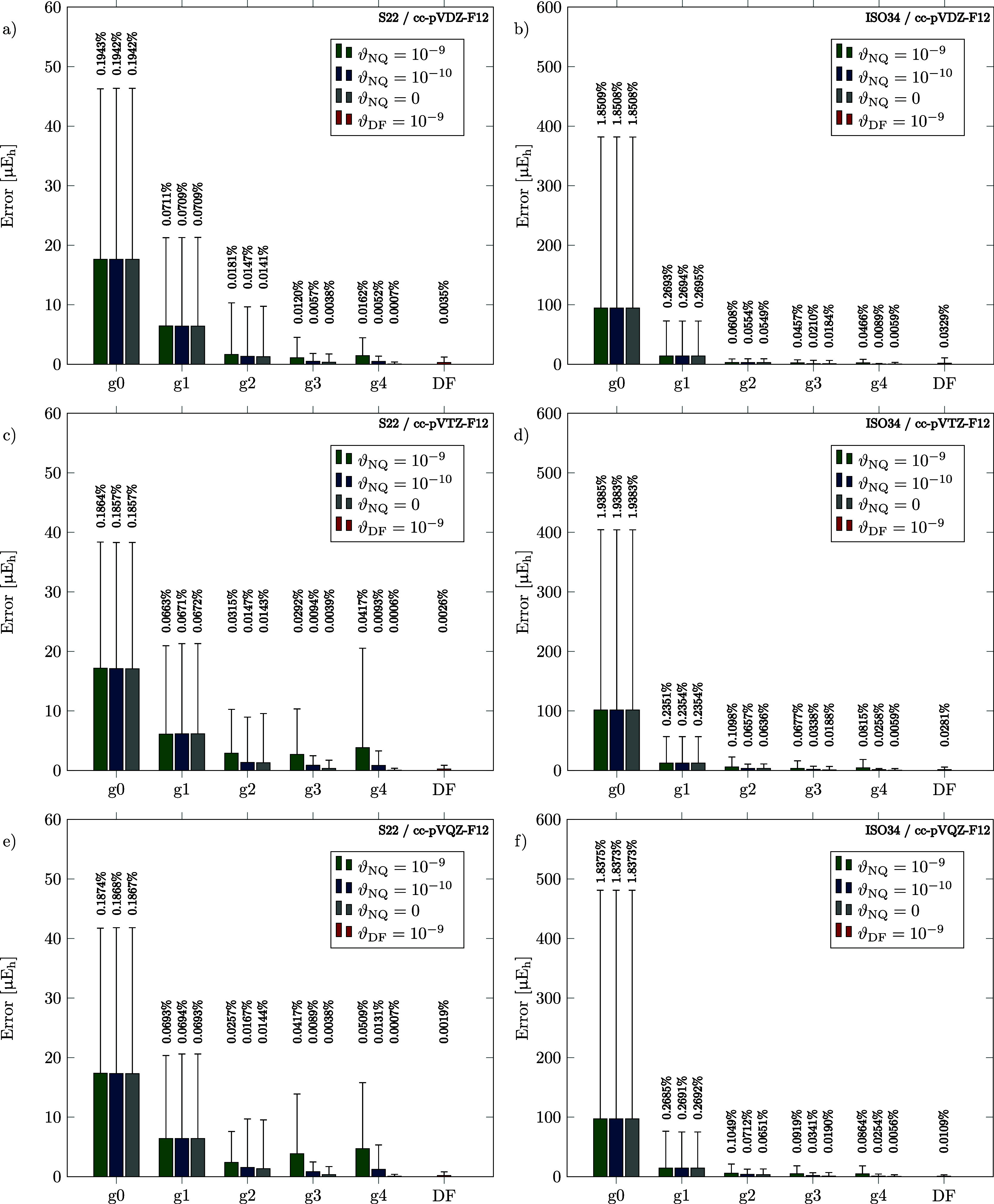
Mean absolute errors (MAE), maximum absolute errors (MAX), and MAEs relative to the average reference DF-MP2 + F12 noncovalent interaction or isomerization energy (MAE/AVG in %) for the S22 (a,c,e) and ISO34 (b,d,f) test sets, respectively. Results are shown for DF/NQ/CABS-RI with various grid sizes (g0 to g4) and thresholds ϑ_NQ_, as well as for DF/CABS-RI with ϑ_DF_ = 10^–9^. Data are given for cc-pVDZ-F12 (a,b), cc-pVTZ-F12 (c,d), and cc-pVQZ-F12 (e,f) basis set combinations.

Generally, NQ/DF/CABS-RI delivers high precision already for medium-sized grids. For example, the mean absolute errors for a g2 grid are below 6 μE_h_ (0.004 kcal/mol) for the S22 and ISO34 test sets. As expected, systematically increasing the grid size further improves the precision, with results converging toward the numerically exact limit. Here, we follow our previously developed screening protocol in ref 82, designed to avoid under- and overscreening of the AO 3c1e integrals. For larger grids (g3 and g4), a small intentional error persists, increasing slightly from double- to quadruple-ζ basis due to the larger number of screened basis functions per atom. With a screening threshold of ϑ_NQ_ = 10^–9^, mean errors range from about 1 to 5 μE_h_, with maximum deviations of around 20 μE_h_ (0.013 kcal/mol). For ϑ_NQ_ = 10^–10^, mean errors are lower, ranging from 0.5 to 1.5 μE_h_, with maximum deviations of around 10 μE_h_ (0.006 kcal/mol).

Our NQ/DF/CABS-RI approach maintains a grid-defined level of precision that is largely independent of basis set size with consistent mean absolute and percentage errors. While the F12 correction diminishes with increasing basis set size, the absolute grid error remains essentially constant, whereas the contribution of the DF-MP2 correlation energies increases. The conventional DF/CABS-RI approach yields nearly error-free results that converge toward the exact limit as both the basis set and the DF basis set are increased, albeit at a substantially higher computational cost. Notably, the finest grids (g3 and g4) match or even exceed the precision of the conventional DF approach, particularly when a sufficiently tight screening threshold is applied. To further investigate the precision for larger molecular systems, Figure 2 presents results for the L7 interaction energy test set, which comprises structures of up to 112 atoms. As expected, errors decrease with increasing grid size, although absolute deviations are somewhat larger than those for the smaller S22 set.

**Figure 2:**
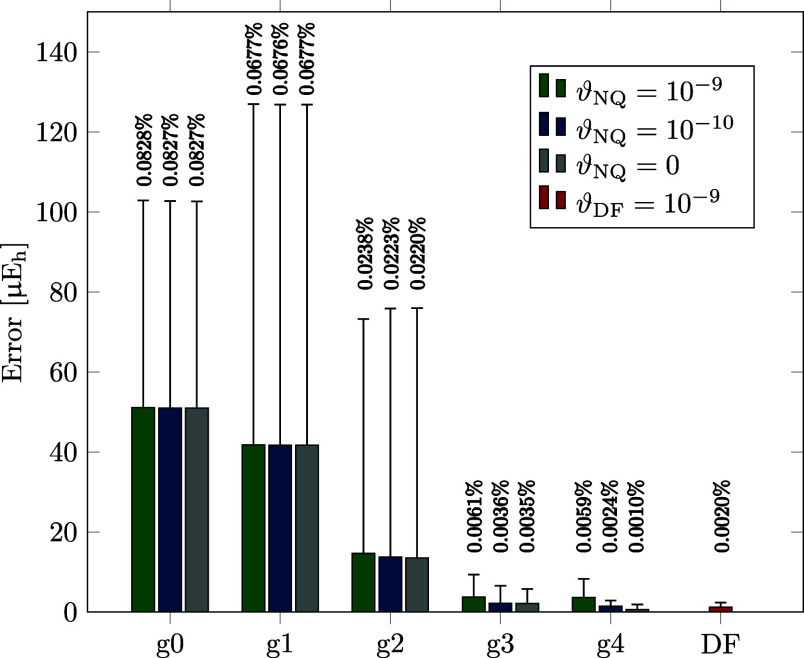
Mean absolute errors (MAE), maximum absolute errors (MAX), and MAEs relative to the average reference DF-MP2 + F12 noncovalent interaction energy (MAE/AVG in %) for the L7 test set. Results are shown for DF/NQ/CABS-RI with various grid sizes (g0–g4) and thresholds ϑ_NQ_, as well as for DF/CABS-RI with ϑ_DF_ = 10^–9^ for a cc-pVDZ-F12 basis set combination.

Across all test sets, the g2 grid serves as our standard choice, yielding negligible errors with maximum deviations below 80 μE_h_ (0.05 kcal/mol) and often substantially smaller. The g1 grid remains suitable for many applications with maximum errors below 130 μE_h_ (0.08 kcal/mol). Even the coarsest g0 grid, while providing less accurate estimates for isomerization energies, remains useful for interaction energies in larger systems with percentage errors around 0.1%. For comparison, the largest member of the L7 test set, which features the highest number of basis functions, shows an error of 0.11% with a g0 grid, whereas DF-MP2 without the F12 correction introduces an error of 4.63%.

In general, the target precision of NQ/DF/CABS-RI calculations can be adjusted to the specific application, enabling an optimal balance between numerical precision and computational efficiency. To avoid introducing additional errors, we chose a ϑ_NQ_ = 10^–10^ screening threshold for 3c1e integrals in our performance tests of NQ/DF/CABS-RI.

### 4.2 Performance Comparison

Figure 3a,b depict the scaling behavior of Algorithm 1 and Algorithm 2 for different grid sizes (g0 to g3) using linear glycine chains. Algorithm 1 exhibits slightly lower efficiency for small system sizes than Algorithm 2, which can be attributed to the enhanced locality of the ${\mathrm{F̂}}_{12}^{2}$ operator. For the largest systems, the scaling exponents of Algorithm 2 remain around 1.3–1.4 due to the growing contribution of additional terms arising from the commutator relation. Overall, both algorithms achieve near-linear scaling, with exponents around 1.4 or 1.1, respectively. For the corresponding direct terms, we anticipate that combining RI-J with the J-engine ansatz^100,113^ will yield the best performance, which, however, lies beyond the scope of the present work.

**Figure 3:**
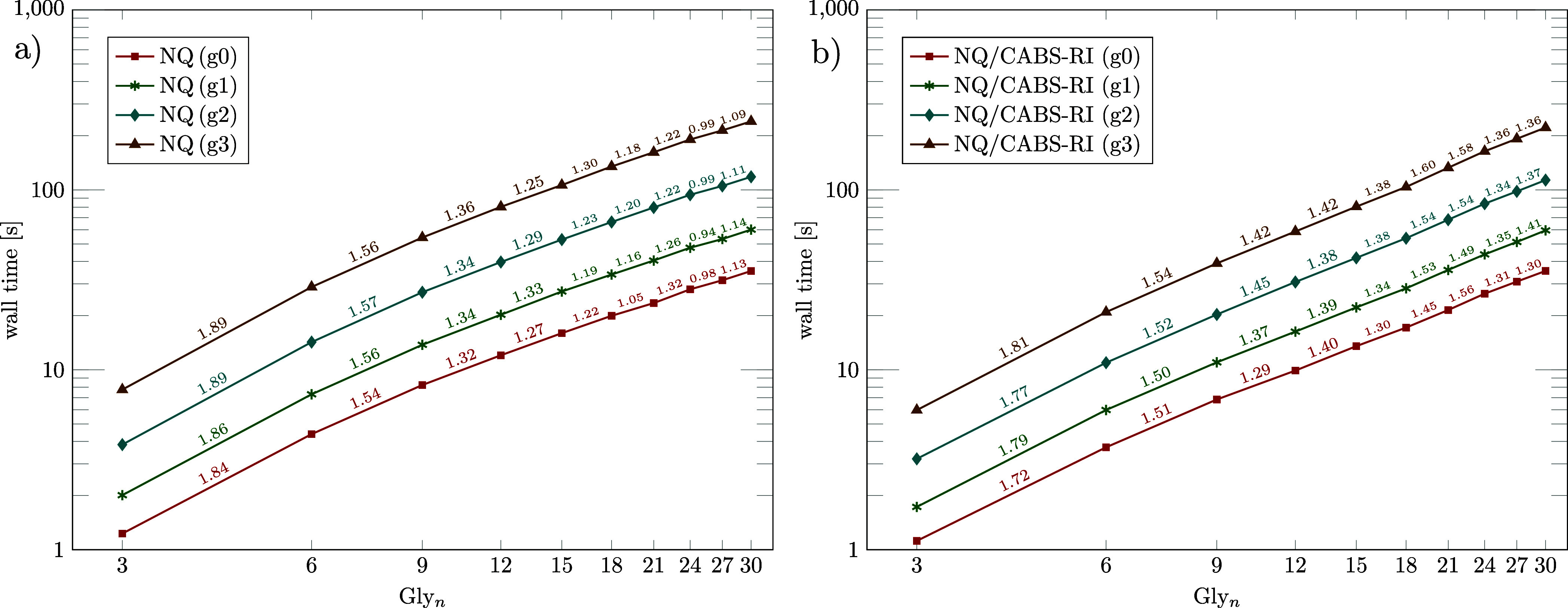
Log–log plot of the wall times and the corresponding scaling exponents between consecutive structures for Algorithm 1 (a) and Algorithm 2 (b) (ϑ_NQ_ = 10^–10^), using multiple grid sizes for linear glycine chains (Gly_*n*_, *n* ∈ {3, 6, 9, 12, 15, 18, 21, 24, 27, 30}) with a cc-pVDZ-F12 basis set combination.

Since Algorithm 3 encompasses the most computationally demanding part of the whole explicitly correlated F12 correction, we place special emphasis on investigating its scaling behavior and performance relative to the classical DF/CABS-RI approach. To enable a unified comparison across different quantum chemical codes, we report the total number of TFLOPs per structure and per calculation for the rate-determining $O(M^{4})$ steps of our novel NQ/DF/CABS-RI method, alongside the corresponding timings. For the classical DF/CABS-RI approach, performance is evaluated under the assumption of peak hardware efficiency, counting the total number of TFLOPs for the performance-critical $O(M^{4})$ and $O(M^{5})$ scaling steps. This framework provides a quantitative basis to assess the efficiency and scaling of our new approach relative to established methodologies. The theoretical peak double-precision (FP64) performance of the two AMD EPYC 9334 processors used in this study, with a total of 64 cores and an aggregate theoretical peak parallel clock frequency of 2.7 GHz, can be estimated as follows. Each core supports two FMA units, with each AVX-512 FMA executing eight FP64 operations per instruction. At the maximum boost frequency, the processors lead to a theoretical peak performance of

64×2.7GHz×2FMA/core×8FP64/FMA≈2.76TFLOPs 72

This value represents a theoretical upper bound for the achievable double-precision throughput on this processor. In our CPU benchmarks, we employed matrix–matrix multiplication of 10,000 × 10,000 matrices using highly optimized BLAS routines (DGEMM). The matrices were allocated once and subsequently reused with random values over multiple iterations so that no additional costs for memory allocation, data copying, or management occurred during the measurement. This achieved a performance of about 2.35 TFLOPs/s, corresponding to roughly 85% of the theoretical peak performance. Since no practical implementation can surpass the performance of pure DGEMM and most fall noticeably short, we take this value as an idealized reference for all DF/CABS-RI timings. To analyze the performance of Algorithm 3 (NQ/DF/CABS-RI) for different grid sizes, we again considered linear glycine chains, with results summarized in Figure 4 for cc-pVDZ-F12 (a,c,e) and cc-pVTZ-F12 (b,d,f) basis set combinations. Panels (a) and (b) present log–log plots of wall times together with the scaling exponents between consecutive structures. Here, the empirical scaling behavior of Algorithm 3 approaches $O(M^{4})$ with system size *M*, starting from an effective scaling of approximately $O(M^{3})$ for small systems where the construction of the three-center one-electron integrals has a strong influence on performance. Panels (c) and (d) compare the theoretical floating-point operation counts for the rate-determining steps per structure (TFLOPs) for NQ/DF/CABS-RI as well as DF/CABS-RI. The speedups shown in panels (e) and (f) are obtained by comparing measured NQ/DF/CABS-RI timings against an idealized perfectly performing DF/CABS-RI implementation (DGEMM efficiency). As expected, smaller grids lead to better performance and an earlier break-even point between DF/CABS-RI and NQ/DF/CABS-RI. Moreover, NQ/DF/CABS-RI gains additional efficiency for larger basis sets since the number of grid points remains essentially constant and thus scales like a fixed index, which benefits the lower-scaling NQ-based formulation.

**Figure 4:**
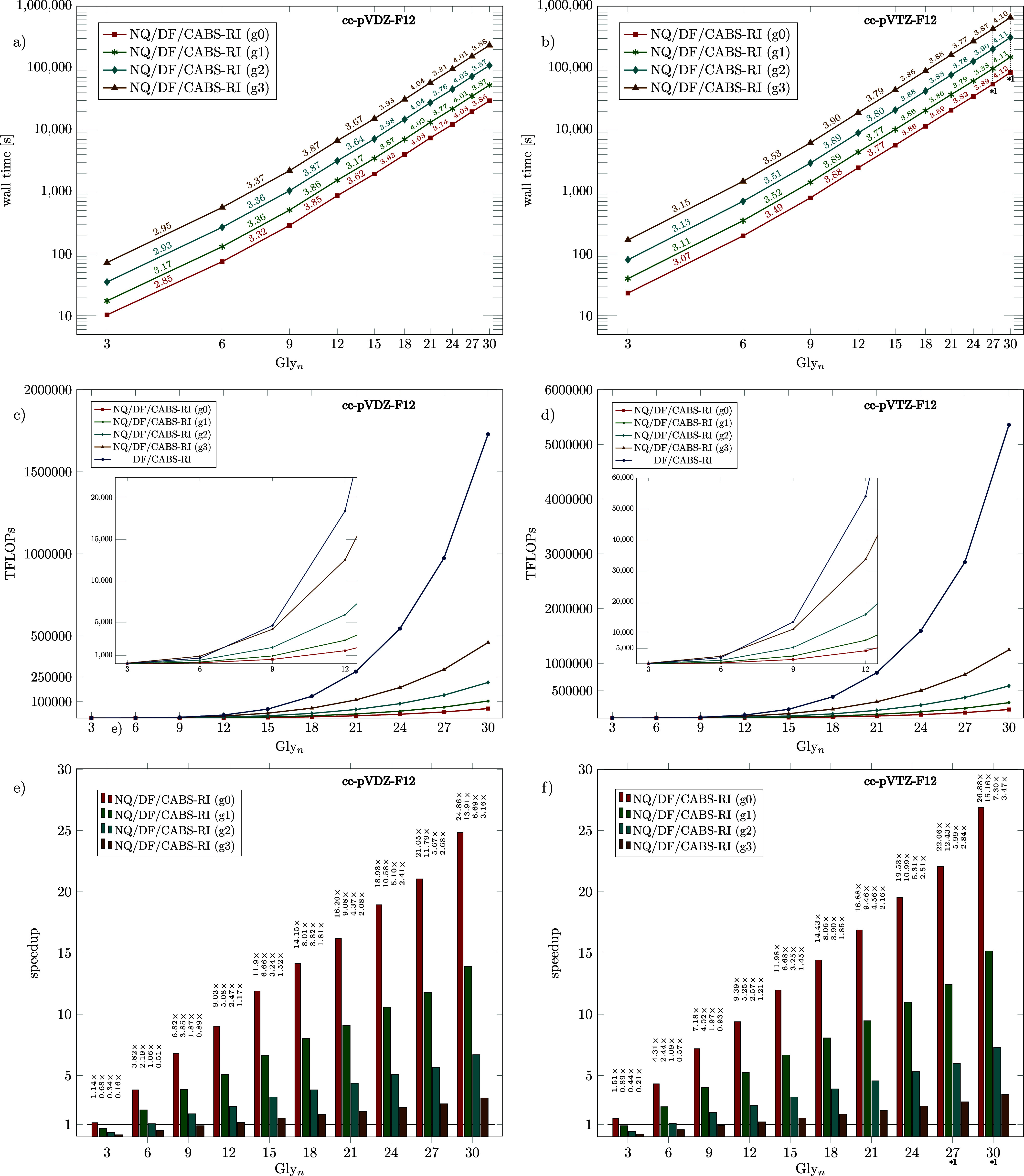
Comparison of Algorithm 3 (NQ/DF/CABS-RI with ϑ_NQ_ = 10^–10^, using multiple grid sizes) and DF/CABS-RI for linear glycine chains (Gly_n_, *n* ∈ {3, 6, 9, 12, 15, 18, 21, 24, 27, 30}): Log–log plots of wall times and scaling exponents between consecutive structures (a,b), comparisons of floating point operations for the rate-determining steps per structure (TFLOPs) (c,d), and corresponding speedups for NQ/DF/CABS-RI (e,f) using cc-pVDZ-F12 (a,c,e) and cc-pVTZ-F12 (b,d,f) basis set combinations.

Overall, NQ/DF/CABS-RI exhibits clear advantages starting from Gly_9_ (C_18_H_29_N_9_O_10_), achieving roughly twice the speed of DF/CABS-RI. This advantage increases with system size, reaching up to 6.7× speedup for the g2 grid with a double-ζ basis. For the largest triple-ζ calculations (Gly_27_ and Gly_30_), computational times were carefully estimated based on FLOP counts, as the required RAM exceeded available memory. For Gly_30_, this results in estimated speedups ranging from 3.5× to 26.9×, depending on grid size. RAM limitations for larger systems can be addressed through extended batching, which entails computing the 3c1e MO integrals *N*_batch_^3c1e^ as an additional cost. Further optimization of the batching procedure beyond Algorithm 3, such as Lagrangian-based minimal-overhead batching,^114^ would be possible but lies outside the scope of the present work.

Figure 5 highlights the performance of NQ/DF/CABS-RI relative to DF/CABS-RI for two representative real-world examples: (a) daptomycin, an antibiotic drug of last resort, and (b) a carbon nanotube with a highly delocalized electronic structure. For daptomycin, speedups ranging from 21.69 to 2.74 are achieved across different grid sizes, where our NQ/DF/CABS-RI code shows around 70% of the theoretical maximum hardware efficiency (2.76 TFLOPs/s). Generally, for systems with a significant HOMO–LUMO gap, further performance gains of NQ/DF/CABS-RI appear feasible by introducing some form of localized MOs and corresponding screening, for example, through a pair natural orbital approach,^44,58,115^ distance-dependent MO screening via integral partition bounds,^91^ or a hybrid strategy. This perspective is motivated by the observation that, for this structure, even in the MO picture, roughly 50% of the 3c1e integral values fall below 10^–12^ for both the ${\mathrm{F̂}}_{12}$ and ${ĝ}_{12}$ operators.

**Figure 5:**
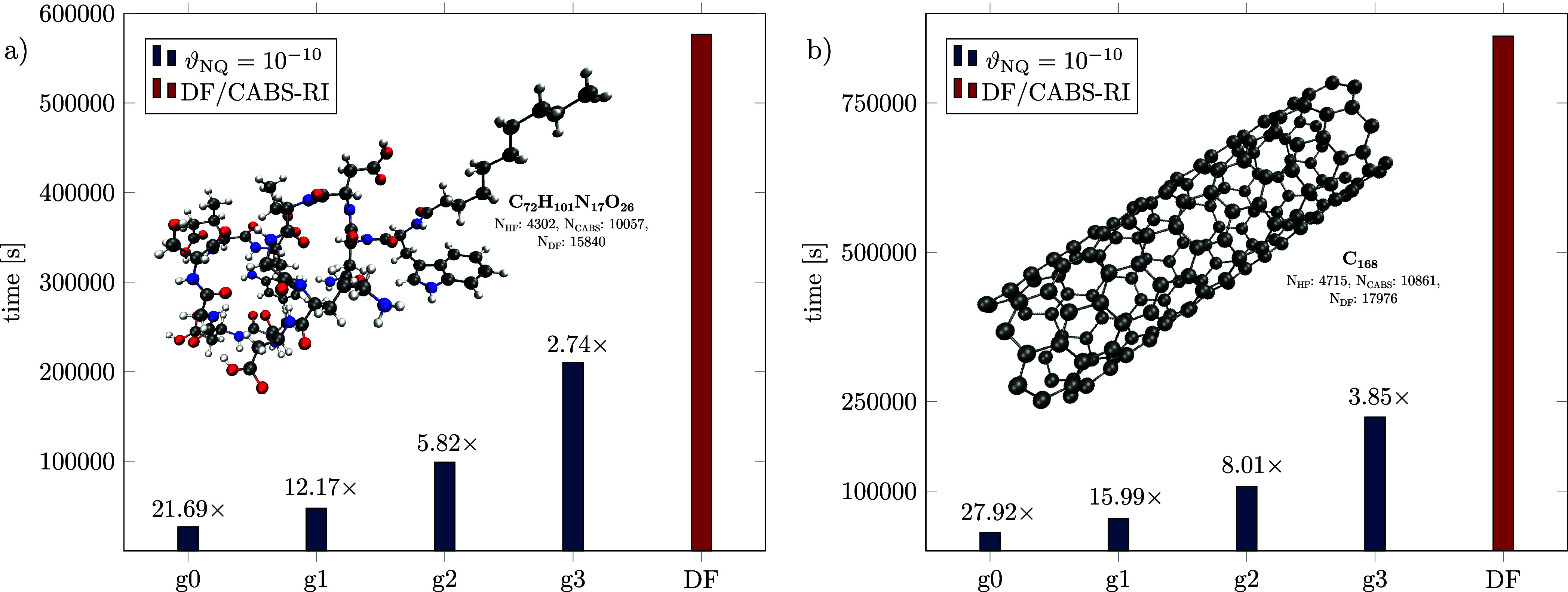
NQ/DF/CABS-RI and DF/CABS-RI timings and corresponding speedups for (a) daptomycin (C_72_H_101_N_17_O_26_) and (b) a C_168_ nanotube, using multiple grid sizes and ϑ_NQ_ = 10^–10^ with a cc-pVDZ-F12 basis set combination (HF/CABS-RI/DF).

For the computed nanotube, localized MO-based integral screening might compromise accuracy due to the strong delocalization of the electronic structure. In this case, NQ/DF/CABS-RI itself drastically reduces the computational time relative to DF/CABS-RI by factors of 27.92 (g0), 15.99 (g1), 8.01 (g2), and 3.85 (g3). A g2 grid provides a particularly favorable balance, lowering the cost by roughly one order of magnitude while maintaining high precision. Although the nanotube involves a comparable number of basis functions as daptomycin, it achieves better performance because of the more favorable ratio of active orbitals to numerical grid points. This demonstrates the efficiency and robustness of NQ/DF/CABS-RI, especially for systems with strongly delocalized electronic structures.

Figure 6 outlines the relative computational cost for an RI-MP2-F12 calculation of the C_168_ nanotube, including SCF and DF-MP2 steps, using DF/CABS-RI and NQ/DF/CABS-RI with g1, g2, and g3 grids. While the cost of the SCF (∼15 min) and DF-MP2 (∼4.5 h) calculations remains overall the same, the DF/CABS-RI route requires an additional 9.97 days for the F12 correction, whereas the hybrid NQ/DF/CABS-RI approach requires only approximately 1.25 days for a g2 grid. Considering the full calculation (SCF + DF-MP2 + F12), excluding the exchange-type terms involving ${\mathrm{F̂}}_{12}$ and ${ĝ}_{12}$ , both methods required approximately 10 h. The computation of the direct-type intermediates accounts for only a minor fraction of the total cost, typically being significantly less demanding than the construction of the required 3c2e integrals. For our NQ ansatz, the additional cost for these exchange-type terms (Algorithm 3) amounts to 14.9 h for g1, 29.9 h for g2, and 62.2 h for g3, respectively. Although Algorithm 3 still dominates the computation, its cost is substantially lower than the 239.4 h required by DF/CABS-RI. Regarding the individual steps of Algorithm 3, the computational time for the construction of AO 3c1e integrals becomes negligible for these system sizes due to effective screening, while the AO to MO transformation of the 3c1e integrals accounts for roughly 10–15% (△). The contraction of the 3c2e integrals with the MO quantities on the grid dominates the overall workload, contributing approximately 65–70% (□), including the formally most expensive step, followed by the final contraction with the 3c1e integrals, which contributes around 20% (○). Following our approach and considering even larger systems, the total cost for the F12 correction will eventually drop below that of the corresponding DF-MP2 calculation when the same basis set is used, potentially rendering CBS extrapolation methods unnecessary.

**Figure 6:**
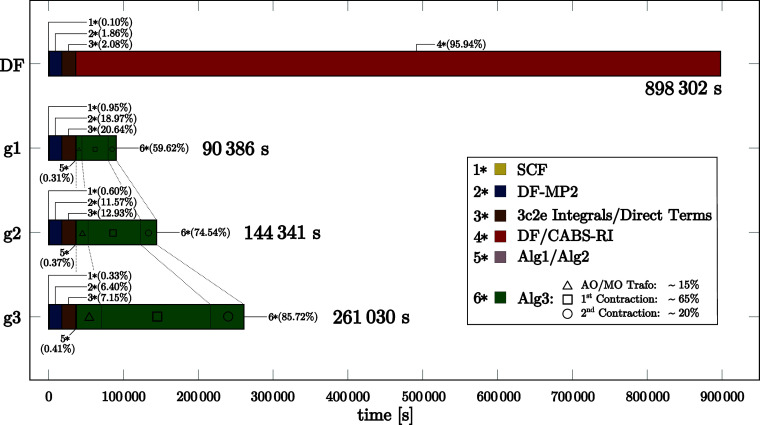
Absolute and relative computational costs for SCF + DF-MP2 + F12 calculations of a C_168_ nanotube, comparing DF/CABS-RI (estimated via DGEMM efficiency) with NQ/DF/CABS-RI using g1, g2, and g3 grids, including a detailed cost breakdown, all computed with the cc-pVDZ-F12 basis set combination.

## 5 Conclusion

We have introduced a novel and efficient framework for treating exchange-type contributions within the explicitly correlated F12 correction to second-order Mo̷ller–Plesset perturbation theory. By employing numerical quadrature, both the formal and the observed empirical scaling with respect to the system size *M* are rigorously reduced from $O(M^{5})$ to $O(M^{4})$ . The approach is rooted in an approximation-free formulation of the strong-orthogonality operator *Q̂*_12_, which enables a fundamentally different treatment of the key exchange-type intermediates V , X , and B . In this reformulation, exchange-type four-center two-electron (4c2e) and six-center three-electron (6c3e) integrals can be evaluated with high efficiency in the AO picture and exhibit linear scaling with the system size by means of the NQ/CABS-RI approach. The remaining products of 4c2e integrals are computed using a novel MO scheme that combines numerical quadrature with density fitting, denoted as NQ/DF/CABS-RI. Moreover, the resulting expressions require fewer CABS-RI insertions, with indices spanning the full orbital space being contracted at an early stage of the evaluation. For both NQ/CABS-RI and NQ/DF/CABS-RI, we have developed efficient algorithms that enable the systematic contraction of all terms while exploiting shared subspaces and intermediate quantities.

Benchmarks on several representative test sets demonstrate that our approach attains accuracies comparable to those of the conventional DF/CABS-RI ansatz, with the precision systematically controlled by the quadrature grid size. Even for large molecules, a modest g2 grid yields mean absolute deviations to the explicitly correlated correction below 0.01 kcal/mol and maximum errors under 0.05 kcal/mol, which ensures reliable results for both noncovalent interaction and isomerization energies. Overall, NQ/DF/CABS-RI enables a flexible balance between numerical precision and efficiency.

We further investigated the scaling behavior of the proposed methods using linear glycine chains. The results demonstrate near linear scaling for our NQ/CABS-RI ansatz, resulting in a negligible computational cost, even for large systems. Generally, the largest computational effort arises from the products of 4c2e integrals, which can be efficiently evaluated with $O(M^{4})$ scaling using DF/NQ/CABS-RI. To enable a unified comparison across different software packages, we take as reference an idealized DF/CABS-RI implementation achieving the efficiency of highly optimized BLAS-3 matrix–matrix (DGEMM) operations on the target hardware, considering only the number of floating-point operations in the rate-determining contractions and ignoring memory management. This choice is intentionally conservative and likely underestimates the speedups achievable in practice. In this comparison, for a g2 grid and molecules of approximately 40 second-period atoms (C, N, O, F) and corresponding H atoms, the NQ/DF/CABS-RI approach achieves more than twice the computational speed. For larger systems, the speedup increases significantly; e.g., a chain of 30 glycine monomers using a cc-pVTZ-F12 basis set combination exhibits speedups ranging from 3.5× to 26.9×, depending on grid size. For real-world examples, speedups of roughly one order of magnitude are achieved for the rate-determining steps with virtually no loss of numerical precision. This effect is particularly pronounced for systems with strongly delocalized electronic structures, where the computational time for the total F12 correction can be reduced from 10.6 to 1.5 days. In general, NQ/DF/CABS-RI substantially narrows the gap in computational cost between the explicitly correlated F12 correction and the corresponding conventional DF-MP2 calculation using the same basis set. Future work will focus on developing an efficient screening strategy based on localized MOs to further reduce the computational cost.

## Supplementary Material



## Notes

Detailed data on NQ/DF/CABS-RI benchmark results (S22, S66, ISO34) (PDF)
