# Supplementary material for: Formulation of an Efficient 𝒪 (M 4)-Scaling Explicitly Correlated MP2-F12 Correction by Combining Numerical Quadrature with Density Fitting and CABS-RI
Source: J Chem Theory Comput. 2026 Mar 2;22(6):2808–24. doi: 10.1021/acs.jctc.5c01874 (PMC13019631; doi:10.1021/acs.jctc.5c01874)
Supplement: Supplementary file 1 [file ct5c01874_si_001.pdf]

Formulation of an Efficient $\mathcal{O}(M^4)$-Scaling Explicitly Correlated MP2-F12 Correction by Combining Numerical Quadrature with Density Fitting and CABS-RI

Supporting Information

Lars Urban,[†,‡] Henryk Laqua,[†] Travis H. Thompson,[†] and Christian Ochsenfeld[*,†,‡]

†*Chair of Theoretical Chemistry, Department of Chemistry, University of Munich (LMU), D-81377 Munich, Germany*

‡*Max Planck Institute for Solid State Research, D-70569 Stuttgart, Germany*

E-mail: christian.ochsenfeld@uni-muenchen.de

1 Absolute Energies

To illustrate the contribution of the exchange-type F12 correction to the overall correlation energy, Table S1 reports the mean exchange energies $E^{3^*\mathrm{C}}_{\mathrm{F12,exch}}$ for DF/NQ/CABS-RI and DF/CABS-RI and their ratios relative to the direct contribution ($E^{3^*\mathrm{C}}_{\mathrm{F12,direct}}$), the total F12 correction ($E^{3^*\mathrm{C}}_{\mathrm{F12}}$), and the total correlation energy (DF-MP2 + F12) for the L7 benchmark set (Ref. 111 in main text). As expected from perturbation theory, the exchange corrections are positive (destabilizing), partially canceling approximately 26.5% of the negative (stabilizing) direct contributions. The exchange term accounts for roughly 36% of the net F12 correction, while the total F12 correction contributes approximately 16.4% to the overall correlation energy. Notably, these ratios remain virtually constant across all grid sizes, demonstrating the robustness of the NQ/DF/CABS-RI approach.

Table S1: Mean exchange correlation energies [$\mathrm{mE_h}$] and corresponding ratios [%] for the L7 benchmark set employing NQ/DF/CABS-RI with various grid sizes (g0–g4, $\vartheta_{\mathrm{NQ}} = 10^{-10}$) and DF/CABS-RI ($\vartheta_{\mathrm{IPB}} = 10^{-9}$) for the cc-pVDZ-F12 basis set combination. Reference values (grid-independent): $E_{\text{DF-MP2}} = \mathbf{-7575.729}$ $\mathrm{mE_h}$, $E_{\mathrm{F12,direct}} = \mathbf{-2025.493}$ $\mathrm{mE_h}$.

Method	$E^{3^*\mathrm{C}}_{\mathrm{F12,exch}}$	$\left\lvert\frac{\mathrm{Exch}}{\mathrm{Direct}}\right\rvert$	$\left\lvert\frac{\mathrm{Exch}}{\mathrm{F12}}\right\rvert$	$\left\lvert\frac{\mathrm{F12}}{\mathrm{Corr_{tot}}}\right\rvert$
g0	537.486	26.536	36.121	16.417
g1	537.276	26.526	36.102	16.419
g2	537.301	26.527	36.104	16.419
g3	537.259	26.525	36.100	16.419
g4	537.247	26.524	36.099	16.419
DF	537.770	26.550	36.147	16.415

Figure S1 illustrates the effect of employing NQ/DF/CABS-RI and DF/CABS-RI on the precision of DF-MP2 + F12 overall correlation energies for the L7 benchmark set using the cc-pVDZ-F12 basis set combination. The figure shows mean absolute errors (MAE), maximum absolute errors (MAX), and mean absolute errors normalized to the average reference energy (MAE/AVG) for different grid sizes and screening thresholds ϑ_{NQ}.

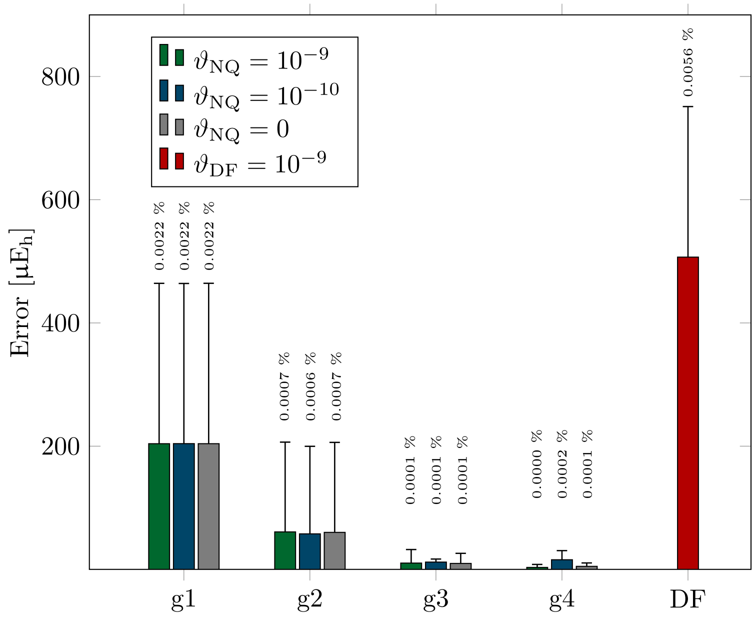

Figure S1: Mean absolute errors (MAE), maximum absolute errors (MAX), and MAEs relative to the average reference absolute energy (MAE/AVG in %) for the L7 test set. Results are shown for NQ/DF/CABS-RI with various grid sizes (g1–g4) and thresholds ϑ_{NQ}, as well as for DF/CABS-RI with $\vartheta_{DF} = 10^{-9}$. Errors were evaluated against a g7 grid ($\vartheta_{NQ} = 0$) reference.

As expected, the error decreases with increasing grid size (g0 results were excluded to improve the visualization). Generally, a g2 grid already provides excellent precision with mean errors of roughly 0.04 kcal/mol, delivering reliable results even for the large system sizes considered. The observed differences in overall energies between DF/CABS-RI and the NQ/DF/CABS-RI reference (using a converged g7 grid) stem from the different mathematical approximations inherent to each approach. Density fitting introduces a systematic error through the incompleteness of the auxiliary basis set. While this error is highly consistent and largely cancels in relative energies (such as non-covalent interactions or isomerization energies), it manifests as a constant shift in absolute energies. In contrast, NQ/DF/CABS-RI evaluates certain components of the exchange-type intermediates directly in real space via numerical quadrature, thereby reducing the reliance on auxiliary basis functions for these terms. Consequently, the two methods converge to slightly different absolute energies, though both provide comparable precision for chemically relevant energy differences, as demonstrated by the benchmark results presented in this work.

2 Isomerization and Non-Covalent Interaction Energies

Table S2: Mean absolute errors [μE_h] (MAEs), max. absolute errors [μE_h] (MAX), and MAEs relative to the average reference RI-MP2 + F12 non-covalent isomerization energy [%] for the **ISO34** test set (Ref. 110 in main text) employing NQ/DF/CABS-RI with various grid sizes ($g0$-$g4$) and thresholds ϑ_{NQ} and DF/CABS-RI ($\vartheta_{IPB} = 10^{-9}$) for different cc-pVXZ-F12 (X = D, T, Q) basis set combinations. Errors were evaluated against a g7 grid ($\vartheta = 0$) reference.

Method		cc-pVDZ-F12			cc-pVTZ-F12			cc-pVQZ-F12		
Grid/DF	ϑ	MAE	MAX	$\frac{\text{MAE}}{\text{AVG}}$	MAE	MAX	$\frac{\text{MAE}}{\text{AVG}}$	MAE	MAX	$\frac{\text{MAE}}{\text{AVG}}$
g0	10^{-9}	94.382	381.816	1.8509	101.543	404.424	1.9385	97.226	481.028	1.8375
	10^{-10}	94.380	381.814	1.8508	101.534	404.286	1.9383	97.216	481.098	1.8373
	0	94.379	381.812	1.8508	101.536	404.284	1.9383	97.215	481.080	1.8373
g1	10^{-9}	13.836	72.728	0.2693	12.363	56.887	0.2351	14.294	76.211	0.2685
	10^{-10}	13.841	72.640	0.2694	12.379	56.749	0.2354	14.325	74.959	0.2691
	0	13.842	72.633	0.2695	12.375	56.723	0.2354	14.332	74.966	0.2692
g2	10^{-9}	3.123	9.025	0.0608	5.769	22.560	0.1098	5.586	21.198	0.1049
	10^{-10}	2.845	9.226	0.0554	3.454	10.752	0.0657	3.792	12.587	0.0712
	0	2.821	9.299	0.0549	3.343	10.896	0.0636	3.468	12.912	0.0651
g3	10^{-9}	2.349	7.448	0.0457	3.560	16.245	0.0677	4.896	18.169	0.0919
	10^{-10}	1.078	6.587	0.0210	1.778	7.016	0.0338	1.815	6.471	0.0341
	0	0.947	6.534	0.0184	0.990	6.753	0.0188	1.012	6.834	0.0190
g4	10^{-9}	2.393	8.389	0.0466	4.287	18.513	0.0815	4.601	18.072	0.0864
	10^{-10}	0.459	1.386	0.0089	1.356	3.332	0.0258	1.354	4.767	0.0254
	0	0.305	3.132	0.0059	0.308	3.175	0.0059	0.300	3.155	0.0056
DF	10^{-9}	1.689	10.896	0.0329	1.480	5.709	0.0281	0.580	3.035	0.0109

Table S3: Mean absolute errors [μE_h] (MAEs), max. absolute errors [μE_h] (MAX), and MAEs relative to the average reference RI-MP2 + F12 non-covalent interaction energy [%] for the **S22** test set (Ref. 109 in main text) employing NQ/DF/CABS-RI with various grid sizes ($g0$-$g4$) and thresholds ϑ_{NQ} and DF/CABS-RI ($\vartheta_{IPB} = 10^{-9}$) for different cc-pVXZ-F12 (X = D, T, Q) basis set combinations. Errors were evaluated against a g7 grid ($\vartheta = 0$) reference.

Method		cc-pVDZ-F12			cc-pVTZ-F12			cc-pVQZ-F12		
Grid/DF	ϑ	MAE	MAX	$\frac{MAE}{AVG}$	MAE	MAX	$\frac{MAE}{AVG}$	MAE	MAX	$\frac{MAE}{AVG}$
g0	10^{-9}	17.628	46.274	0.1943	17.158	38.374	0.1864	17.362	41.734	0.1874
	10^{-10}	17.619	46.357	0.1942	17.095	38.298	0.1857	17.305	41.816	0.1868
	0	17.616	46.358	0.1942	17.093	38.303	0.1857	17.299	41.818	0.1867
g1	10^{-9}	6.436	21.268	0.0711	6.092	20.945	0.0663	6.411	20.335	0.0693
	10^{-10}	6.423	21.291	0.0709	6.167	21.293	0.0671	6.413	20.600	0.0694
	0	6.421	21.320	0.0709	6.169	21.283	0.0672	6.411	20.603	0.0693
g2	10^{-9}	1.636	10.323	0.0181	2.890	10.264	0.0315	2.374	7.594	0.0257
	10^{-10}	1.331	9.662	0.0147	1.350	8.959	0.0147	1.546	9.689	0.0167
	0	1.275	9.747	0.0141	1.310	9.566	0.0143	1.330	9.530	0.0144
g3	10^{-9}	1.087	4.525	0.0120	2.686	10.339	0.0292	3.860	13.872	0.0417
	10^{-10}	0.514	1.813	0.0057	0.868	2.468	0.0094	0.821	2.466	0.0089
	0	0.348	1.738	0.0038	0.355	1.722	0.0039	0.353	1.694	0.0038
g4	10^{-9}	1.469	4.458	0.0162	3.829	20.533	0.0417	4.707	15.781	0.0509
	10^{-10}	0.470	1.366	0.0052	0.857	3.292	0.0093	1.212	5.323	0.0131
	0	0.060	0.383	0.0007	0.056	0.371	0.0006	0.060	0.388	0.0007
DF	10^{-9}	0.314	1.214	0.0035	0.238	0.868	0.0026	0.180	0.800	0.0019

Table S4: Mean absolute errors [μE_h] (MAEs), max. absolute errors [μE_h] (MAX), and MAEs relative to the average reference RI-MP2 + F12 non-covalent interaction energy [%] for the **S66** test set (Ref. 112 in main text) employing NQ/DF/CABS-RI with various grid sizes ($g0$-$g4$) and thresholds ϑ_{NQ} and DF/CABS-RI ($\vartheta_{IPB} = 10^{-9}$) for different cc-pVXZ-F12 (X = D, T, Q) basis set combinations. Errors were evaluated against a g7 grid ($\vartheta = 0$) reference.

Method		cc-pVDZ-F12			cc-pVTZ-F12			cc-pVQZ-F12		
Grid/DF	ϑ	MAE	MAX	$\frac{MAE}{AVG}$	MAE	MAX	$\frac{MAE}{AVG}$	MAE	MAX	$\frac{MAE}{AVG}$
g0	10^{-9}	11.654	40.345	0.1600	10.083	39.472	0.1362	11.541	40.709	0.1547
	10^{-10}	11.649	40.262	0.1600	10.073	39.188	0.1360	11.519	40.571	0.1544
	0	11.650	40.270	0.1600	10.075	39.149	0.1360	11.515	40.556	0.1544
g1	10^{-9}	4.596	22.819	0.0631	4.537	23.084	0.0613	4.595	23.239	0.0616
	10^{-10}	4.589	22.629	0.0630	4.527	22.789	0.0611	4.585	23.123	0.0615
	0	4.587	22.587	0.0630	4.523	22.767	0.0611	4.584	23.130	0.0615
g2	10^{-9}	1.389	8.689	0.0191	2.620	11.173	0.0354	2.618	13.401	0.0351
	10^{-10}	1.141	8.425	0.0157	1.256	7.708	0.0170	1.310	7.545	0.0176
	0	1.151	8.495	0.0158	1.137	8.271	0.0154	1.147	7.952	0.0154
g3	10^{-9}	1.146	5.080	0.0158	2.673	10.359	0.0361	3.495	13.382	0.0469
	10^{-10}	0.391	1.479	0.0054	1.046	6.105	0.0141	0.937	5.523	0.0126
	0	0.173	1.341	0.0024	0.172	1.339	0.0023	0.174	1.299	0.0023
g4	10^{-9}	2.413	18.037	0.0332	4.598	17.132	0.0621	4.805	15.782	0.0645
	10^{-10}	0.364	1.246	0.0050	0.860	3.000	0.0116	0.981	2.931	0.0132
	0	0.056	0.466	0.0008	0.056	0.455	0.0008	0.055	0.457	0.0007
DF	10^{-9}	0.315	1.338	0.0043	0.173	0.774	0.0023	0.151	0.581	0.0020
